# Separation and Characterization of Self-Assembled Nanoparticles from *Rheum palmatum* L.–*Salvia miltiorrhiza* Bunge Extract and Their Renoprotective Effects in Acute Kidney Injury

**DOI:** 10.3390/antiox15040491

**Published:** 2026-04-15

**Authors:** Jing Yang, Chenghong Li, Huaqiao Tang, Xue Xia, Yuanhang Chen, Maixun Zhu, Gang Ye, Fei Shi, Wei Zhang, Cheng Lv, Lixia Li, Xun Wang, Yinglun Li, Ling Zhao

**Affiliations:** 1 College of Veterinary Medicine, Sichuan Agricultural University, Chengdu 611130, China; yangjing4@stu.sicau.edu.cn (J.Y.);; 2 Chongqing Academy of Animal Sciences, Rongchang 402460, China; lich@cqaa.cn (C.L.);; 3 State Key Laboratory of Swine and Poultry Breeding Industry, College of Animal Science and Technology, Sichuan Agricultural University, Chengdu 611130, China

**Keywords:** acute kidney injury, self-assembled nanoparticles, *Rheum palmatum* L.–*Salvia miltiorrhiza* pair, renoprotection, apoptosis

## Abstract

Acute kidney injury (AKI) presents a critical clinical challenge due to its rapid progression and lack of effective targeted therapies. The herbal combination of rhubarb and Salvia miltiorrhiza, a cornerstone of Traditional Chinese Medicine (TCM) for renal protection, shows promise, yet its bioactive components and mode of action remain incompletely understood. This study identifies and characterizes inherent nanoscale entities from this herbal pair as a novel nanotherapeutic platform. Self-assembled nanoparticles (designated RSNPs) were isolated from the ethanol extract via differential centrifugation. Comprehensive characterization revealed that RSNPs form stable nanostructures through spontaneous self-assembly, primarily driven by supramolecular interactions (e.g., π-π stacking and hydrogen bonding). UPLC-MS/MS quantification confirmed the co-assembly of multiple bioactive constituents within RSNPs. Network pharmacology and molecular docking initially predicted their synergistic action on AKI-related pathways. In a cisplatin-induced murine AKI model, RSNP administration markedly attenuated renal dysfunction and histopathological damage, mechanistically linked to the mitigation of oxidative stress (e.g., decreased MDA and increased SOD) and inflammation (e.g., downregulated TNF-α and IL-6). In vitro, RSNPs demonstrated enhanced cellular internalization and superior cytoprotection against cisplatin toxicity in renal tubular epithelial cells, significantly reducing apoptosis. These findings unveil that the therapeutic efficacy of the *Rheum palmatum* L.–*Salvia miltiorrhiza* Bunge pair is intrinsically embedded within its nanoscale architecture. RSNPs represent a new class of TCM-derived nanotherapeutics with a well-defined material basis and multimodal mechanisms, offering a promising strategy for AKI treatment.

## 1. Introduction

Acute kidney injury (AKI) poses a growing clinical challenge worldwide, with rising incidence rates and substantial associated mortality creating significant burdens on healthcare systems [1,2]. This condition is characterized by rapid renal function deterioration, primarily driven by interconnected pathological mechanisms including oxidative stress, inflammatory cascades, and programmed cell death, which collectively contribute to tubular damage and functional decline [3,4]. While renal replacement therapy has seen technical advancements, the absence of pharmacological interventions specifically targeting these core pathological processes represents a critical unmet medical need.

Traditional Chinese Medicine (TCM), with its foundation in multi-target therapeutic approaches, has emerged as a promising avenue for AKI management [5,6]. The classic TCM herb pair of rhubarb (Dahuang) and *Salvia miltiorrhiza* (Danshen) has been extensively documented for its synergistic anti-inflammatory and antioxidant properties, demonstrating considerable efficacy in various experimental models of renal injury [7,8]. However, the clinical translation of the crude extract of rhubarb and *Salvia miltiorrhiza* remains constrained by poor oral bioavailability and insufficient mechanistic understanding of its renal protective effects.

The rapidly evolving field of nanomedicine offers transformative potential for overcoming these inherent limitations of natural products [9,10]. Particularly intriguing is the recent recognition that certain phytochemicals can spontaneously self-assemble into carrier-free nanoparticles, a process that enhances bioactivity and improves targeting capability [11,12,13]. We hypothesized that the active constituents within RSE might inherently form such self-assembled nanostructures (RSNPs), potentially serving as integrated therapeutic platforms.

In this investigation, we isolated and characterized RSNPs from RSE, employing comprehensive analytical techniques to decipher their physicochemical properties, chemical composition, and self-assembly mechanisms. We subsequently evaluated the nephroprotective efficacy of RSNPs using a CDDP-induced AKI model and elucidated the underlying mechanisms through an integrated approach combining network pharmacology, molecular docking, and experimental validation. Our findings not only establish RSNPs as a compelling nano-therapeutic candidate for AKI but also provide a conceptual framework for developing next-generation TCM-inspired nanomedicines.

## 2. Materials and Methods

### 2.1. Materials

Cisplatin (CDDP), Tris-HCl buffer, 2,2-Di(4-tert-octylphenyl)-1-picrylhydrazyl (DPPH), and Pyrogallol were purchased from Shanghai Yuanye Bio-Technology Co., Ltd. (Shanghai, China). 2,2′-azinobis (3-ethylbenzothiazoline-6-sulfonic acid) (ABTS) was purchased from Coolaber Biotechnology Co., Ltd. (Beijing, China). Krebs–Ringer buffer was purchased from Solarbio Science & Technology Co., Ltd. (Beijing, China). Assay kits for blood urea nitrogen (BUN), blood creatinine (CRE), catalase (CAT), glutathione (GSH), and superoxide dismutase (SOD) were all purchased from Nanjing Jiancheng Bioengineering Institute (Nanjing, China). Assay kits for tumor necrosis factor-α (TNF-α), interleukin-1β (IL-1β), and interleukin-6 (IL-6) were sourced from Quanzhou Ruixin Biological Technology Co., Ltd. (Quanzhou, China). The 293T human renal tubular epithelial cells were kindly provided by the research team specializing in Traditional Chinese Veterinary Medicine at the College of Veterinary Medicine, Sichuan Agricultural University.

### 2.2. Preparation of Rheum palmatum L.–Salvia miltiorrhiza Extract

The dried materials of rhubarb (*Rheum palmatum* L.; Lixian County, Gansu) and *Salvia miltiorrhiza* (Zhongjiang County, Sichuan) were weighed in a specific ratio (1:1, *w*/*w*), powdered, and macerated in 70% (*v*/*v*) ethanol at room temperature for 1 h. The mixture was then subjected to reflux extraction twice using 70% ethanol at a solid-to-solvent ratio of 1:10 (*w*/*v*), each extraction lasting 3 h. The resulting filtrates were combined and concentrated using a rotary evaporator (model RE-3000A; Shanghai Yarong, Shanghai, China) to obtain the rhubarb–Salvia extract (RSE) solution, with a final concentration equivalent to 0.2 g crude drug per mL.

### 2.3. Separation of the Self-Assembled Nanoparticles in RSE

Self-assembled nanoparticles (RSNPs) were isolated from RSE through differential centrifugation, exploiting differential sedimentation rates arising from distinct density and hydrodynamic characteristics [14]. Briefly, 2 mL of RSE was initially centrifuged at 3000 rpm for 10 min at 4 °C using a refrigerated high-speed centrifuge (SCILOGEX, CF1524R, Rocky Hill, CT, USA) to remove large particulate matter. The resulting supernatant was subsequently subjected to high-speed centrifugation at 15,000 rpm for 40 min at 4 °C to pellet the nanoscale assemblies. Following supernatant removal, the pellet was resuspended in deionized water, and this purification cycle was repeated three times to eliminate residual soluble contaminants. The final suspension was filtered through a 1 μm syringe filter to remove potential aggregates, yielding purified RSNPs. The nanoparticles were lyophilized and stored at −80 °C for subsequent experimental use.

### 2.4. The Characterization Methods of RSNPs

The hydrodynamic diameter, zeta potential, and polydispersity index (PDI) of RSNPs were determined employing a Zetasizer Pro system (Malvern Panalytical, Malvern, UK). Samples were appropriately diluted and equilibrated at 25 °C for 2 min before analysis. Dynamic light scattering (DLS) measurements were performed to assess particle size distribution and PDI, while zeta potential was evaluated using the phase analysis light scattering (M3-PALS) technique. Samples were diluted 1:100 with deionized water and equilibrated at 25 °C for 2 min before measurement. Each sample was measured in triplicate. Morphological characterization was carried out using a JEM-F200 transmission electron microscope (JEOL, Tokyo, Japan). Specimens were prepared by depositing 10 μL of sample suspension onto carbon-coated copper grids, allowing 3 min for adsorption, followed by negative staining with 10 μL of phosphotungstic acid (2%, *w*/*v*) for 1 min. After air-drying, samples were imaged at an acceleration voltage of 120 kV. Structural analysis was conducted using a Nicolet iS50 FTIR spectrometer (Thermo Fisher Scientific, Waltham, MA, USA). Lyophilized samples were mixed with potassium bromide and compressed into transparent pellets. Spectral data were collected in the range of 4000–400 cm^−1^ with 4 cm^−1^ resolution. UV-Vis absorption spectra were acquired using a UV-3600 Plus spectrophotometer (Shimadzu, Tokyo, Japan). Samples were loaded into 1 cm pathlength quartz cuvettes and scanned from 200 to 800 nm, with the corresponding dispersion medium serving as a blank reference.

### 2.5. Systematic Evaluation of the Stability of RSNPs

The stability of RSNPs was systematically evaluated to assess their potential as oral delivery vehicles, with investigations conducted from both temporal and acid-base perspectives [15]. For temporal stability, lyophilized RSNP powder was stored at −80 °C for 1, 3, and 5 days. At each time point, samples were reconstituted and subjected to hydrodynamic diameter measurement to evaluate the physical integrity of the lyophilized formulation during storage. To assess acid-base stability, RSNPs were dispersed in buffer systems at pH 2.2 (simulating gastric fluid) and pH 7.4 (simulating intestinal fluid), followed by incubation at 37 °C for predetermined intervals. Changes in particle size distribution and aggregation behavior were monitored to comprehensively evaluate the bio-interface stability of RSNPs throughout the simulated oral delivery process.

### 2.6. Antioxidant Capacity of RSNPs

#### 2.6.1. ABTS Free Radical Scavenging Capacity

ABTS was dissolved in a 2.45 mM potassium persulfate solution and incubated in the dark at room temperature for 12 h to generate the ABTS radical cation (ABTS•^+^) stock solution at a concentration of 7 mM. The resulting mixture was diluted 20-fold with PBS (0.05 M, pH 7.4) to obtain the working ABTS•^+^ solution. Subsequently, 0.2 mL of each sample solution at various concentrations was combined with 2 mL of the ABTS•^+^ working solution and allowed to react for 5 min at room temperature. The absorbance of the mixture (A_sample_) was then measured at 734 nm [16]. A control was prepared by replacing the sample solution with PBS, and its absorbance (A_control_) was recorded under identical conditions. The ABTS radical scavenging activity was calculated according to the following Equation (1):

(1) $$\begin{array}{c} ABTS\;radical\;scavenging\;activity\left(\%\right)=\left(1-\frac{A_{\mathrm{sample}}}{A_{\mathrm{control}}}\right)\times 100\% \end{array}$$

#### 2.6.2. DPPH Free Radical Scavenging Capacity

DPPH (4 mg) was accurately weighed and dissolved in 100 mL of absolute ethanol to prepare a stock solution, which was stored protected from light at 0–4 °C. To each reaction tube, 1 mL of RSNPs at varying concentrations was introduced, followed by the addition of 4 mL of DPPH working solution. Control samples were prepared by substituting the nanoparticle suspension with an equal volume of absolute ethanol. All mixtures were then incubated in the dark for 30 min, after which the UV absorbance was determined at 517 nm [17]. The DPPH radical scavenging activity was calculated using Equation (2):

(2) $$\begin{array}{c} DPPH\;scavenging\;activity\left(\%\right)=\frac{A_{\mathrm{control}}-A_{\mathrm{sample}}}{A_{\mathrm{control}}}\times 100\% \end{array}$$

where A_control_ is the absorbance of the blank, and A_sample_ is the absorbance of the sample.

#### 2.6.3. •OH Scavenging Capacity

A Fenton reaction system was established to evaluate the hydroxyl radical (•OH) scavenging capacity of RSNPs. Specifically, a 6 mmol/L FeSO_4_ solution was prepared and supplemented with a trace amount of 1 mol/L H_2_SO_4_ to maintain iron in its reduced state. A 6 mmol/L salicylic acid solution was prepared in absolute ethanol, and a 6 mmol/L H_2_O_2_ working solution was freshly diluted from 30% stock. In 10 mL centrifuge tubes, 2 mL of each reagent—FeSO_4_, salicylic acid, and H_2_O_2_—was combined with 2 mL of RSNPs at varying concentrations. After thorough mixing, the reaction mixtures were incubated at 37 °C for 30 min [18]. The absorbance of the hydroxylated salicylic acid complex was measured at 510 nm, and the •OH scavenging rate was calculated using Equation (3):

(3) $$\begin{array}{c} •OH\;scavenging\;activity\left(\%\right)=\frac{A_{\mathrm{control}}-A_{\mathrm{sample}}}{A_{\mathrm{control}}}\times 100\% \end{array}$$

where A_control_ is the absorbance of the blank, and A_sample_ is the absorbance of the sample.

#### 2.6.4. •O_2_^−^ Scavenging Capacity

Pyrogallol (0.0315 g) was accurately weighed and dissolved in distilled water, then transferred to a 100 mL volumetric flask and diluted to volume to prepare a 2.5 mM stock solution, which was freshly prepared before each experiment. For the assay, 5 mL of Tris-HCl buffer (50 mM, pH 8.2), 2 mL of sample solutions at varying concentrations, and 0.1 mL of the 2.5 mM pyrogallol solution were combined and thoroughly mixed. The reaction mixture was allowed to stand for 40 min at room temperature, after which the reaction was terminated by adding two drops (approximately 0.1 mL) of 8 mol/L HCl solution [19]. The absorbance was measured at 320 nm, and the superoxide anion radical (•O_2_^−^) scavenging activity was calculated using Equation (4):

(4) $$\begin{array}{c} {{•O}_{2}}^{-}\;scavenging\;activity\left(\%\right)=\frac{A_{\mathrm{control}}-A_{\mathrm{sample}}}{A_{\mathrm{control}}}\times 100\% \end{array}$$

where A_control_ is the absorbance of the blank, and A_sample_ is the absorbance of the sample.

#### 2.6.5. Comparative Analysis of Free Radical Scavenging Capacity

This study compared the free radical scavenging capacity of RSE and its nanoparticle formulation RSNPs. Stock solutions of both compounds were prepared at 400 μg/mL in aqueous medium. Radical scavenging activity was evaluated against both ABTS and DPPH radicals according to established protocols. All measurements were performed in triplicate with three independent experimental replicates. Data are presented as mean ± SD.

### 2.7. UPLC-MS/MS Analysis

Aliquots (100 μL) of homogenized samples were transferred to 1.5 mL microcentrifuge tubes, vortexed for 30 s, and supplemented with 300 μL of 95% methanol. After thorough mixing, the mixtures were centrifuged at 17,000× *g* for 10 min at 20 °C. The resulting supernatants were collected for subsequent chromatographic analysis. Separation was performed on an ACQUITY UPLC HSS T3 column (Waters Technologies Co., Ltd., Shanghai, China, 1.8 μm, 2.1 × 100 mm) maintained at 40 °C. An injection volume of 4 μL was employed with a mobile phase comprising 0.1% (*v*/*v*) formic acid in water (A) and acetonitrile (B). Elution was carried out using a linear gradient from 5% to 95% B over 30 min at a constant flow rate. Analysis was conducted using an AB 5600 Triple TOF mass spectrometer (AB Sciex LLC, Shanghai, China) equipped with an electrospray ionization (ESI) source. The ion source parameters were optimized as follows: nebulizer gas (GS1) 60 psi, auxiliary gas 60 psi, curtain gas 35 psi, source temperature 550 °C, and ion spray voltage 5500 V (positive mode) or −4500 V (negative mode). Data acquisition was performed in both positive and negative ionization modes to ensure comprehensive metabolite detection. Chromatographic and spectral data were processed using Analyst^®^ TF 1.8 software (AB Sciex, Shanghai, China) for peak integration and compound identification. Compound identification was performed based on accurate mass measurements (error < 5 ppm), retention time, fragmentation patterns, and comparison with the PubChem, MassBank, and in-house databases. Where available, reference standards were used for confirmation.

### 2.8. The Rat Everted Intestinal Sac Experiment

All animal procedures, including mouse AKI modeling and rat intestinal absorption studies, were approved by the Animal Welfare and Ethics Committee of Sichuan Agricultural University (Approval No. 20240113) and conducted in accordance with institutional guidelines. The intestinal absorption characteristics of cryptotanshinone and rhein were evaluated using an everted gut sac model. Male Sprague–Dawley rats (6 weeks old, 230–270 g, SPF Biotechnology Co., Ltd. (Beijing, China)) were fasted for 12 h and sacrificed to obtain duodenal, jejunal, and ileal segments. The intestinal segments (10 cm) were flushed with ice-cold Krebs–Ringer buffer, everted using a glass rod, and ligated at both ends to form sealed sacs. The gut sacs were equilibrated in oxygenated Krebs–Ringer buffer (95% O_2_/5% CO_2_) at 37 °C for 3 min [20]. RSNPs nanoparticle solutions (5, 7.5, and 10 mg/mL) and RSE (10 mg/mL) were used as test formulations. Each sac was filled with 1.0 mL blank buffer in the serosal compartment and immersed in 50 mL drug solution in the mucosal compartment. Serosal samples (200 μL) were collected at predetermined time points (0–240 min), with an equal volume of fresh buffer replenished after each sampling.

After centrifugation at 12,000× *g* for 10 min, 50 μL of supernatant was analyzed by HPLC. Chromatographic separation was achieved using a Supersil ODS2 column (Dalian Elite Analytical Instruments Co., Ltd., Dalian, China, 2.1 × 100 mm, 1.8 μm) with a mobile phase of 0.02% phosphoric acid-acetonitrile (80:20, *v*/*v*) at 1.0 mL/min. Detection was performed at 280 nm with the column maintained at 25 °C.

### 2.9. Network Pharmacology-Based Investigation of RSNPs

#### 2.9.1. Screening of Active Components and Target Prediction

Bioactive compounds in RSNPs were systematically profiled using a multi-database screening strategy. UPLC-MS/MS analysis informed initial screening through TCMSP (https://tcmsp-e.com/tcmsp.php, accessed on 11 February 2024; OB ≥ 30% and DL ≥ 0.18), HERB (http://herb.ac.cn/, accessed on 11 February 2024; OB ≥ 30%), and ETCM (http://www.tcmip.cn/ETCM/, accessed on 11 February 2024; DL ≥ 0.18), with deduplicated results forming a preliminary compound set. Further refinement via Swiss ADME (http://www.swissadme.ch/, accessed on 12 February 2024) required high gastrointestinal absorption and fulfillment of ≥3 drug-likeness rules (Lipinski, Ghose, Veber, Egan, Muegge). The final library integrated compounds met either standard OB/DL thresholds or Swiss ADME criteria.

For target identification, canonical SMILES strings from PubChem (https://pubchem.ncbi.nlm.nih.gov/, accessed on 13 February 2024) were processed through SwissTargetPrediction (https://www.swisstargetprediction.ch/, accessed on 13 February 2024), retaining targets with probability ≥ 0.1. These were combined with targets from CTD (https://ctdbase.org/, accessed on 3 February 2024) to construct a comprehensive network for mechanistic analysis.

#### 2.9.2. Acquisition of AKI-Associated Targets

AKI-associated targets were identified through integrated multi-database mining and transcriptomic profiling. OMIM (https://omim.org/, accessed on 13 February 2024) and GeneCards (https://www.genecards.org/, accessed on 13 February 2024) were queried using “AKI” and “acute kidney injury”, retaining GeneCards targets with relevance scores > 10 and combining them with OMIM-derived targets after deduplication.

Parallel analysis of the GEO database (https://www.ncbi.nlm.nih.gov/geo/, accessed on 14 February 2024) with “acute kidney injury” retrieved human transcriptomic datasets. Differential expression analysis using the limma R package (version 3.52.2, *p* < 0.05, |log_2_FC| > 1) identified significantly dysregulated genes, with results visualized through ggplot2 and ComplexHeatmap packages (version 2.12.1), generating volcano plots and hierarchical clustering heatmaps.

#### 2.9.3. Compound–Target Interactions and PPI Network

AKI therapeutic targets were identified by intersecting targets from GEO and specialized databases, retaining only those present in ≥2 independent sources. These targets were integrated with RSNP targets to define potential intervention targets. Compound–target–disease relationships were established using Cytoscape 3.10.1, while compound prioritization employed the TOPSIS algorithm with multi-criteria assessment. After normalizing target counts and chromatographic peak areas with equal weighting (0.5 each), quantitative proximity scores (C_i_) were derived from Euclidean distance calculations for final ranking [21].

PPI networks were generated using STRING (https://cn.string-db.org/, accessed on 13 February 2024) under stringent parameters (Homo sapiens, confidence ≥ 0.40), complemented by Metascape (https://metascape.org, accessed on 13 February 2024) validation. Network topology analysis via CytoNCA computed degree, closeness, and betweenness centrality metrics. Targets were ranked by median centrality values, with the top five identified as core therapeutic targets.

#### 2.9.4. GO and KEGG Enrichment Analysis

To delineate the biological functions and pathways modulated by RSNPs in AKI intervention, the intersecting targets were analyzed through functional enrichment using the DAVID platform (https://david.ncifcrf.gov/home.jsp, accessed on 13 February 2024) under the parameter settings: “official gene symbol,” “gene list,” and “Homo sapiens.” Gene Ontology (GO) and Kyoto Encyclopedia of Genes and Genomes (KEGG) pathway analyses were performed with a significance threshold of *p* < 0.01. Enrichment results were visualized via the Microbioinformatics online toolkit, while molecular pathway diagrams were generated using the pathview package in R, with significantly enriched genes highlighted in red.

### 2.10. Molecular Docking Verification

Three-dimensional structures of core target proteins were acquired from the UniProt database (https://www.uniprot.org/, accessed on 13 February 2024) and Protein Data Bank (http://www.rcsb.org/, accessed on 13 February 2024) for use as molecular receptors. Concurrently, two-dimensional structures of principal RSNPs constituents were retrieved from PubChem to serve as ligands. Molecular docking simulations were conducted using MOE 2019 to quantify binding affinity between receptors and ligands. The resulting docking poses were subsequently visualized and analyzed through the computational tools integrated in the MOE 2019 software suite.

### 2.11. Molecular Dynamics (MD) Simulations

**Figure 1:**
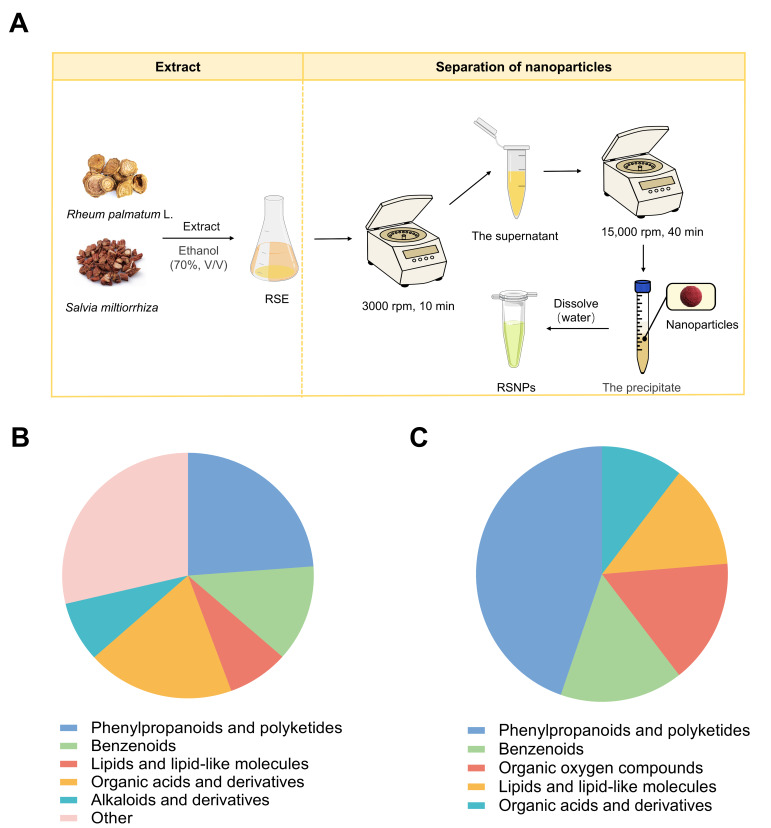

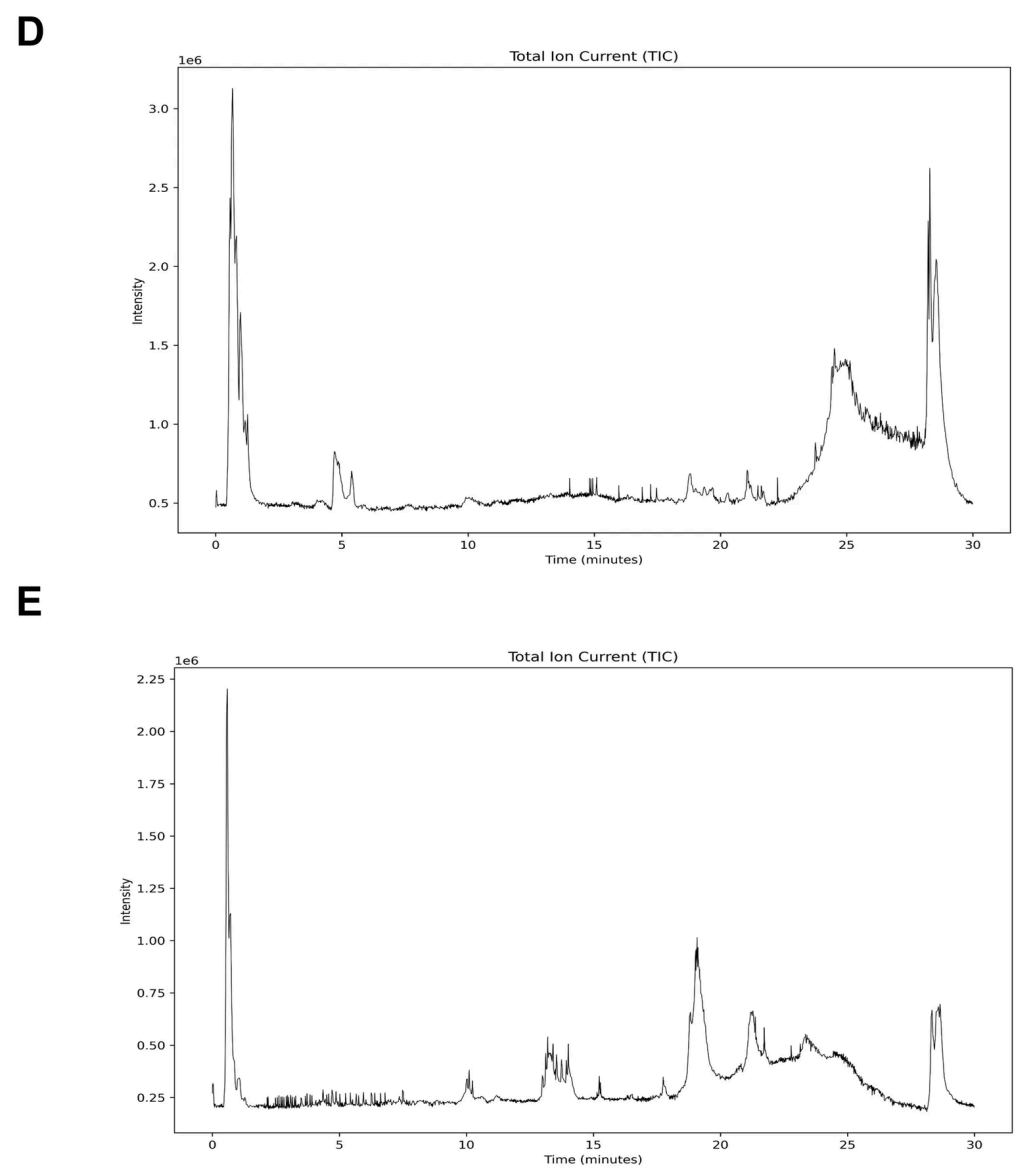
Preparation, phytochemical composition, and chromatographic profiling of *Rheum palmatum* L.–*Salvia miltiorrhiza* self-assembled nanoparticles (RSNPs). (**A**) Schematic illustration of the isolation and purification process of RSNPs. (**B**,**C**) Classification of phytochemicals identified in RSNPs by UPLC-MS/MS analysis: Compound distribution by chemical class detected in (**B**) positive and (**C**) negative ionization modes, respectively. (**D**,**E**) Representative base peak intensity (BPI) chromatograms of RSNPs acquired in (**D**) positive and (**E**) negative ionization modes.

A cubic simulation box with a volume of 10 nm^3^ was constructed, containing 10 rhein molecules and 38 cryptotanshinone molecules, as illustrated in Figure 1. Molecular interactions within the system were described using the OPLS-AA force field, while atomic partial charges were derived using the RESP method. Prior to the simulation, the system underwent energy minimization using the conjugate gradient (CG) algorithm to eliminate unphysical atomic overlaps and reduce the initial potential energy. Subsequently, a 100 ns self-assembly process was carried out under the canonical (NVT) ensemble. During the simulation, the temperature was maintained using the Nosé–Hoover thermostat with a time step of 1 fs. Non-bonded interactions were treated with a cut-off distance of 12.0 Å, while long-range electrostatic interactions were computed using the particle–particle/particle–mesh (PPPM) method. All simulations were performed using the LAMMPS software package (2022 version).

### 2.12. Animal Experiments

Male ICR mice (6 weeks old, 18–22 g) were obtained from Chengdu Vitalriver Experimental Animal Co., Ltd. (Chengdu, China). The animals were randomly divided into five groups (*n* = 10): Normal control (saline), Model control (saline), RSE (1.0 g/kg, crude drug equivalent), Low-dose RSNPs (L-RSNPs, 0.5 g/kg), and High-dose RSNPs (H-RSNPs, 1.0 g/kg). All groups except the Normal control received a single intraperitoneal injection of CDDP (15 mg/kg) on day 4. Three days post-CDDP administration, the mice were anesthetized, underwent terminal blood collection, and were euthanized by cervical dislocation for kidney tissue harvest in subsequent experimental investigations.

#### 2.12.1. Assessment of Renal Function in Mice

The collected kidneys were processed for organ weight measurement and gross morphological assessment. The kidney index was determined as the ratio of kidney weight to body weight (×100%). For serum isolation, whole blood samples were incubated at 37 °C for 30 min, followed by centrifugation at 3500 r/min for 10 min. The obtained serum was stored at −80 °C and subsequently analyzed for BUN and Scr concentrations using commercial assay kits following the manufacturers’ instructions.

#### 2.12.2. Evaluation of Inflammation and Oxidative Stress

Renal tissues (0.1 g) were homogenized in ice-cold normal saline at a 1:9 (*w*/*v*) ratio to prepare 10% tissue homogenates. The homogenates were centrifuged at 3500 r/min for 10 min, and the resulting supernatants were collected for subsequent analysis. The levels of CAT, SOD, and GSH in renal homogenates were measured using specific assay kits according to the manufacturers’ protocols. Concurrently, the concentrations of IL-1β, IL-6, and TNF-α were determined employing commercial ELISA kits following the manufacturers’ instructions.

#### 2.12.3. Histological Analysis

Collected kidney tissues were gently perfused with normal saline, and surface moisture was removed using filter paper. Representative tissue sections were immersion-fixed in 4% paraformaldehyde and subsequently embedded in paraffin blocks. Thin sections (5 μm) were prepared using a microtome, followed by deparaffinization in xylene and graded ethanol dehydration. The sections were then processed through standard histological staining protocols for hematoxylin and eosin (H&E), periodic acid–Schiff (PAS), and Masson’s trichrome. Histopathological assessment was conducted by examining five randomly selected fields per section at 400× magnification using bright-field microscopy. Tubular injury was quantified using a semi-quantitative scoring system based on the percentage of cortical area exhibiting characteristic pathological features: 0 (≤15%), 1 (16–30%), 2 (31–45%), 3 (46–60%), 4 (61–75%), and 5 (≥76%).

### 2.13. Cell Evaluation of RSNPs

Human embryonic kidney 293T cells were cultured in DMEM supplemented with 10% fetal bovine serum (FBS), 100 μg/mL streptomycin, and 100 U/mL penicillin, and maintained at 37 °C in a humidified 5% CO_2_ incubator. Following a 24 h incubation period in 96-well plates, cells were exposed to fresh medium containing RSNPs at concentrations ranging from 5 to 640 μg/mL for an additional 24 h. Cell viability was quantified using the Cell Counting Kit-8 (CCK-8, Dojindo Molecular Technologies Co., Ltd., Shanghai, China) assay according to the manufacturer’s protocol. In parallel experiments, the potential cytotoxic effects of cisplatin (CDDP) at concentrations of 4–256 μg/mL on 293T cells were evaluated using the same methodology. Based on the resultant cytotoxicity profiles, optimal concentrations of CDDP and RSNPs were determined for subsequent experimental investigations.

#### 2.13.1. Protective Effects of RSNPs Against Cellular Injury

In 96-well plates, 293T cells were preconditioned with serially diluted RSNPs for 24 h prior to a 24 h CDDP challenge. Cellular viability was determined by incubating with CCK-8 reagent for 1 h and measuring absorbance at 450 nm using a microplate reader.

For biomarker quantification, cells were pretreated with RSNPs at indicated concentrations before being subjected to CDDP exposure, with parallel controls receiving 8 μg/mL CDDP alone. After 24 h incubation, culture supernatants were harvested and analyzed for LDH release, NO accumulation, and GSH content using corresponding commercial assay kits following manufacturers’ guidelines.

#### 2.13.2. Anti-Inflammatory Capabilities

293T cells were cultured in 6-well plates for 24 h. Following this, the cells were pretreated with RSNPs for 24 h and subsequently stimulated with 8 μg/mL CDDP for an additional 24 h period. The concentrations of key inflammatory mediators (IL-1β, IL-6, and TNF-α) in the culture supernatant were precisely quantified using specific ELISA kits (Thermo Fisher Scientific Inc., Waltham, MA, USA), following a standardized protocol.

#### 2.13.3. Quantification of Apoptotic Cells by Flow Cytometry

Cell suspensions were prepared at 10^6^ cells/mL and aliquoted into 100 μL samples. After centrifugation at 300× *g* for 5 min, pellets were resuspended in 100 μL of 1× binding buffer. Sequential staining was performed with 5 μL Annexin V-FITC (10 min, dark) followed by 10 μL propidium iodide (5 min, dark). Cells were then resuspended in 400 μL phosphate-buffered saline and immediately analyzed using a CytoFlex flow cytometer (Beckman Coulter, Brea, CA, USA). Data analysis employed standard gating strategies to quantify distinct apoptotic populations.

#### 2.13.4. RNA Extraction and Real-Time PCR

Total RNA was extracted from 293T specimens using a commercial RNA isolation kit, with subsequent quantification of RNA concentration and assessment of purity performed by UV spectrophotometry. Reverse transcription of purified RNA to cDNA was carried out using a standardized cDNA synthesis kit, with resulting cDNA aliquots preserved at −20 °C for future analysis. Quantitative real-time polymerase chain reaction (qRT-PCR) assays were conducted using the LightCycler 480II Master mixture (Roche, Indianapolis, IN, USA) in 10 μL reaction volumes (Table 1). Thermal cycling parameters followed the blastaq™ 2 × qPCR MasterMix manufacturer’s protocol: initial enzyme activation at 95 °C for 3 min, followed by 40 cycles of denaturation at 95 °C for 15 s and combined annealing/extension at 60 °C for 1 min.

### 2.14. Statistical Analysis

Data are expressed as mean ± standard deviation (SD) from at least three independent replicates (n ≥ 3). Comparisons between two groups were performed using an unpaired two-tailed Student’s *t*-test. Comparisons among multiple groups were analyzed by one-way analysis of variance (ANOVA) followed by Tukey’s post hoc test for multiple comparisons. GraphPad Prism 8.0 was used for all analyses, with *p* < 0.05 considered statistically significant.

## 3. Results

### 3.1. Confirmation of Compounds in the RSNPs

According to the Chinese Pharmacopoeia (2020 Edition), the contents of emodin and chrysophanol in Rhei Radix et Rhizoma, as well as tanshinone IIA and salvianolic acid B in Salviae Miltiorrhizae Radix et Rhizoma, were determined by HPLC prior to the experiment. The results demonstrated that all batches met the pharmacopoeial standards. RSE was prepared following a standardized procedure using equal masses (1:1) of both medicinal herbs extracted with 70% ethanol. RSNPs were then isolated from the extract through differential centrifugation. This process involved sequential centrifugation at low and high speeds to remove particulate matter of different sizes, after which the resulting nanoparticles were resuspended in aqueous medium (Figure 1A).

Compositional analysis using UPLC-MS/MS allowed the identification of 101 distinct phytochemical constituents in RSNPs (S1). Among these, 63 compounds were detected in positive ionization mode and 38 in negative ionization mode. The detailed information of the identified compounds, including retention time, molecular formula, and molecular mass, is summarized in Table 2 (positive ion mode) and Table 3 (negative ion mode). Structural classification assigned these compounds to five major chemical classes: alkaloids and their derivatives, lipids and lipid-like molecules, phenylpropanoids and polyketides, organic acids and derivatives, and benzenoids (Figure 1B,C). The “benzenoids” group comprises various aromatic subclasses commonly present in *Rheum palmatum* and *Salvia miltiorrhiza*, including anthraquinones (e.g., rhein and emodin), phenolic acids, and flavonoids.

**Table 2:** Information on the positive ion compounds from RSNPs tentatively characterized by UPLC-MS/MS.

NO.	Retention Time (min)	Name	Molecular Formula	Molecular Mass (g/mol)
1	0.70	Hypoxanthine	C_5_H_4_N_4_O	136.11
2	1.02	Putrescine	C_4_H_12_N_2_	88.15
3	19.62	Cryptotanshinone	C_19_H_20_O_3_	296.4
4	21.02	Tanshinone Iia	C_19_H_18_O_3_	294.3
5	0.57	4-methylumbelliferyl glucuronide	C_16_H_16_O_9_	352.29
6	0.70	Tyramine	C_8_H_11_NO	137.18
7	0.54	L-Arginine	C_6_H_14_N_4_O_2_	174.2
8	21.72	Musk ketone	C_14_H_18_N_2_O_5_	294.3
9	20.70	Phthalic anhydride	C_8_H_4_O_3_	148.11
10	29.66	1,2,3-Trihydroxybenzene	C_6_H_6_O_3_	126.11

All reported masses are observed values from UPLC-MS/MS analysis. Theoretical masses are not listed as they are consistent with the molecular formulas provided.

**Table 3:** Information on the negative ion compounds from RSNPs tentatively characterized by UPLC-MS/MS.

NO.	Retention Time (min)	Name	Molecular Formula	Molecular Mass (g/mol)
1	13.31	Sebacic acid	C_10_H_18_O_4_	202.25
2	0.59	5-Demethylnobiletin	C_20_H_20_O_8_	388.4
3	0.62	Geniposide	C_17_H_24_O_10_	388.4
4	17.79	Emodin	C_15_H_10_O_5_	270.24
5	0.59	Turanose	C_12_H_22_O_11_	342.3
6	0.87	Pyroglutamic acid	C_5_H_7_NO_3_	129.11
7	19.39	Deoxycholic acid	C_24_H_40_O_4_	392.6
8	26.15	Embelin	C_17_H_26_O_4_	294.4
9	15.19	Rhein	C_15_H_8_O_6_	284.22
10	0.59	Mannose	C_6_H_12_O_6_	180.16

All reported masses are observed values from UPLC-MS/MS analysis. Theoretical masses are not listed as they are consistent with the molecular formulas provided.

### 3.2. Identification of the Bioactive Components of RSNPs

**Figure 2:**
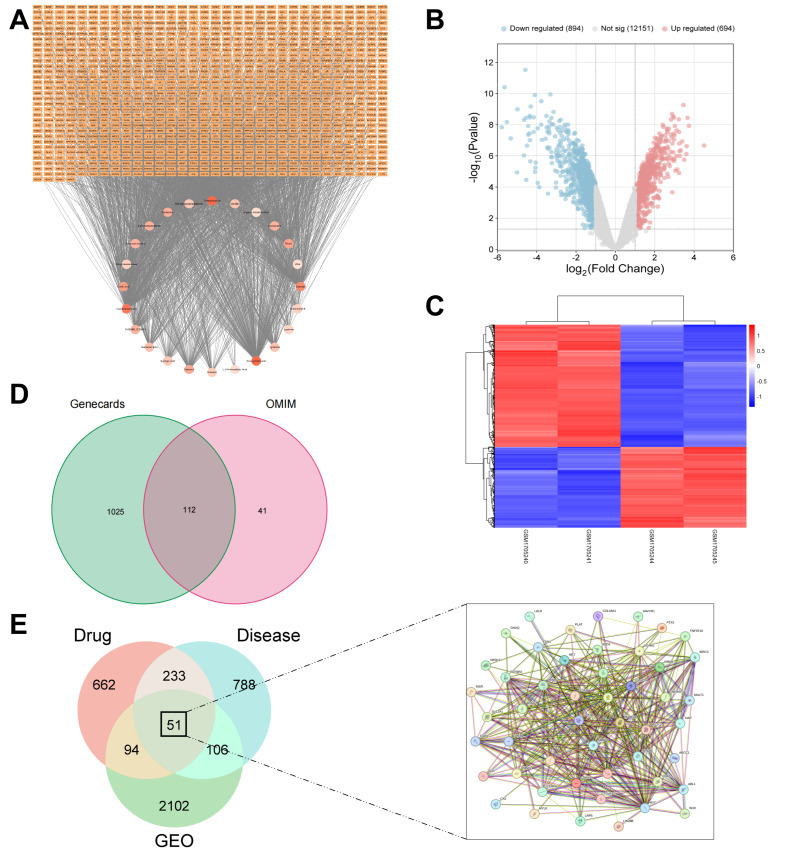
Identification of potential RSNP targets for AKI. (**A**) Compound-target network. The network contains 1052 nodes (compounds and targets) and 2266 edges. Node color denotes topological importance. (**B**) Volcano plot of DEGs. Shows gene expression changes (24h cisplatin vs. control). Red/blue indicate up/down-regulated genes. (**C**) Clustering heatmap of DEGs. Displays gene expression patterns across treatment groups. (**D**) Target source overlap. Venn diagram of AKI targets from GeneCards and OMIM databases. (**E**) Core target identification. Intersection of targets from multiple sources yields 51 core targets.

Through integrated screening of the TCMSP, HERB, and ETCM databases, twenty-five bioactive compounds were identified that concurrently satisfied both OB ≥ 30% and DL ≥ 0.18 criteria. Subsequent evaluation of remaining compounds via Swiss ADME revealed seventeen additional bioactive constituents meeting the established parameters, yielding a total of forty-two characterized bioactive compounds from RSNPs (S1). The compound-target network (Figure 2A) illustrates the complex interactions between RSNPs constituents and potential AKI targets. The top-ranked bioactive compounds based on multi-criteria assessment (TOPSIS analysis), including cryptotanshinone, galangin, and rhein, are listed in Table 4 with their proximity scores and weighted rankings.

**Table 4:** Prioritization and scores of core bioactive compounds.

Rank	Compound	Proximity (C_i_)	Weighted Score
1	Cryptotanshinone	0.901	0.537
2	Galangin	0.783	0.385
3	5-Demethylnobiletin	0.732	0.417
4	Rhein	0.681	0.189
5	Deoxycholic acid	0.645	0.273

### 3.3. Integrated Identification of RSNPs Therapeutic Targets in AKI

Systematic integration of GeneCards and OMIM databases yielded 1178 on-redundant AKI-related targets (Figure 2D). The transcriptomic dataset GSE69644 was retrieved from GEO, containing CDDP-treated human renal proximal tubular epithelial cells (HK-2) across control, 6 h, and 24 h treatments with duplicate replicates. Comparative analysis revealed 2366 differentially expressed genes (1424 upregulated and 942 downregulated) in 24 h CDDP-treated groups versus controls, visualized via a volcano plot and hierarchical clustering (Figure 2B,C). Intersection of multi-source AKI targets (≥2 databases) with RSNPs bioactive compounds identified 51 potential therapeutic targets through computational integration (Figure 2E).

### 3.4. Network Analysis of Core Targets and Functional Modules

**Figure 3:**
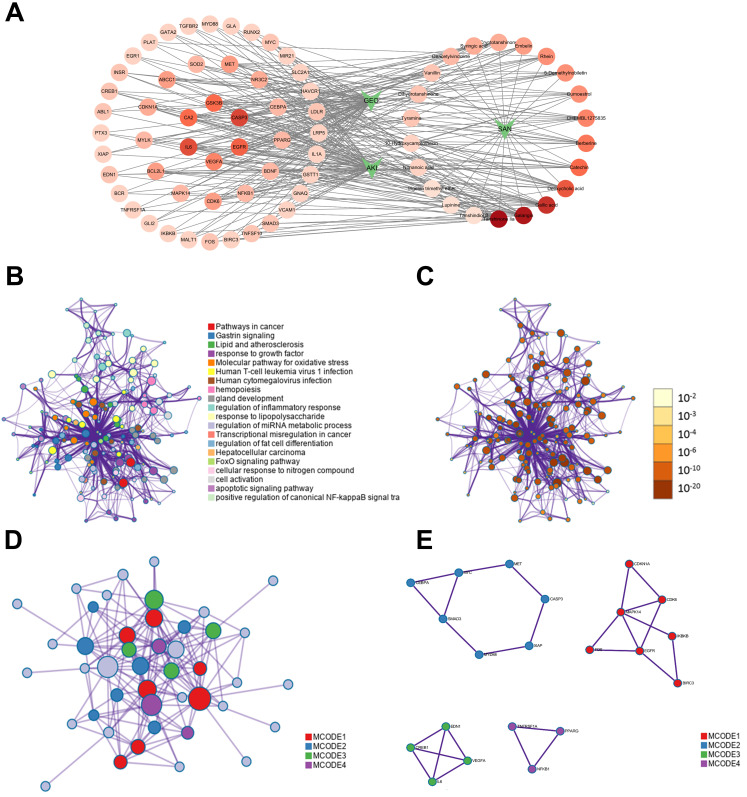

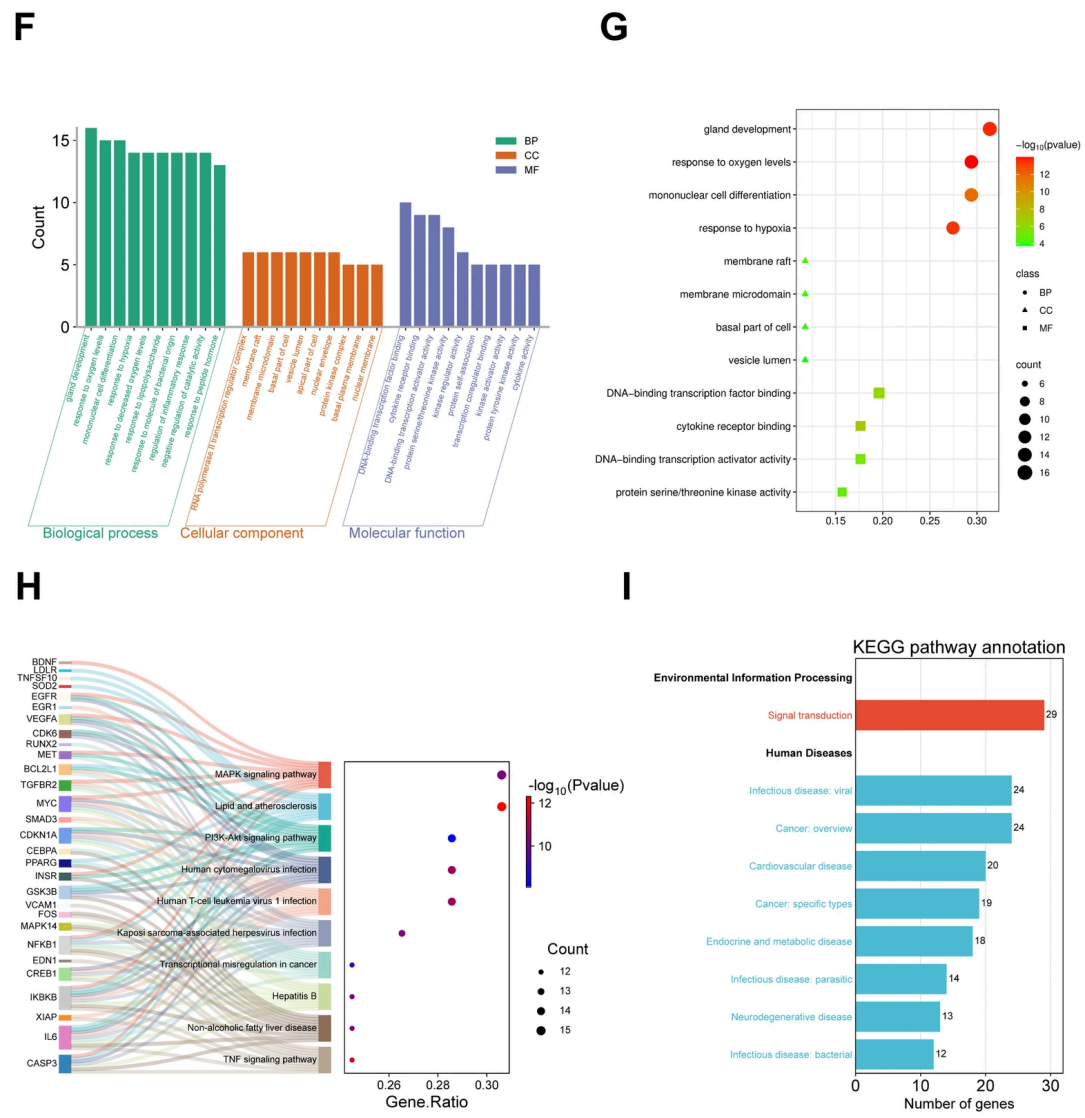
Network and functional module analysis of potential RSNPs targets for AKI. (**A**) The interaction network of overlapping targets from GEO, AKI, and RSNPs. (**B**) Network nodes color-coded by cluster ID, illustrating topological proximity. (**C**) Network nodes color-coded by *p*-value, demonstrating the association between gene count and statistical significance. (**D**,**E**) The protein–protein interaction (PPI) network and its key functional modules, identified by MCODE analysis. (**F**,**G**) Gene Ontology enrichment analysis. (**F**) Bar plot and (**G**) bubble plot display the significantly enriched GO terms of the core targets in biological process, cellular component, and molecular function. (**H**,**I**) KEGG pathway enrichment analysis. (**H**) Bar plot and (**I**) bubble plot show the significantly enriched signaling and disease pathways of the core targets. Pathways in the bubble plot (**I**) are categorized by their functional domains.

Through intersection analysis, we identified 51 overlapping targets shared by AKI pathogenesis and RSNPs bioactive components. A protein–protein interaction network constructed using the STRING database visually represented these targets (Figure 2E) with color-coded gradients indicating their network significance (Figure 3A). Topological analysis via CytoNCA revealed five hub targets-IL-6, CASP3, GSK3B, FOS, and EGFR-based on their superior centrality metrics (Table 5). Functional module analysis using MCODE further delineated four distinct clusters: MCODE1 (red) was enriched in cancer pathways; MCODE2 (blue) connected gastrin signaling to cancer pathways; MCODE3 (green) participated in galanin receptor signaling and humoral homeostasis, while MCODE4 (purple) implicated epigenetic regulation and metabolic disorders. This multi-layer network architecture reveals the complex interplay between oncogenic signaling and metabolic/developmental pathways underlying RSNPs-mediated intervention in AKI (Figure 3B,E).

**Table 5:** Basic topological properties of core regulatory genes.

Uniprot ID	Gene	Degree	Betweenness Centrality	Closeness Centrality
P42574	*CASP3*	20	5.162	1.000
P49841	*GSK3B*	19	4.332	0.952
P05231	*IL6*	20	5.162	1.000
P01100	*FOS*	19	3.589	0.952
P00533	*EGFR*	20	5.162	1.000

### 3.5. GO and KEGG Functional Enrichment

Gene Ontology analysis revealed significant enrichment in several key biological processes, particularly gland development, cellular response to oxygen levels, and mononuclear cell differentiation. For cellular components, notable enrichment was observed in RNA polymerase II transcription regulator complexes, membrane rafts, and membrane microdomains. Assessment of molecular functions indicated predominant involvement in DNA-binding transcription factor binding, cytokine receptor binding, and DNA-binding transcription activator activity (Figure 3F,G).

In parallel, KEGG pathway analysis identified several significantly enriched pathways associated with RSNPs, including MAPK signaling, lipid and atherosclerosis, human T-cell leukemia virus 1 infection, human cytomegalovirus infection, and PI3K-Akt signaling. Core targets such as CASP3, IL-6, IKBKB, and NFKB1 demonstrated dense connectivity and recurrent involvement across multiple pathways (Figure 3H,I). These pathways were further classified into two main categories-environmental information processing and human diseases-reflecting the multi-target mechanism through which RSNPs exert their therapeutic effects against AKI.

### 3.6. Molecular Docking Validation

**Figure 4:**
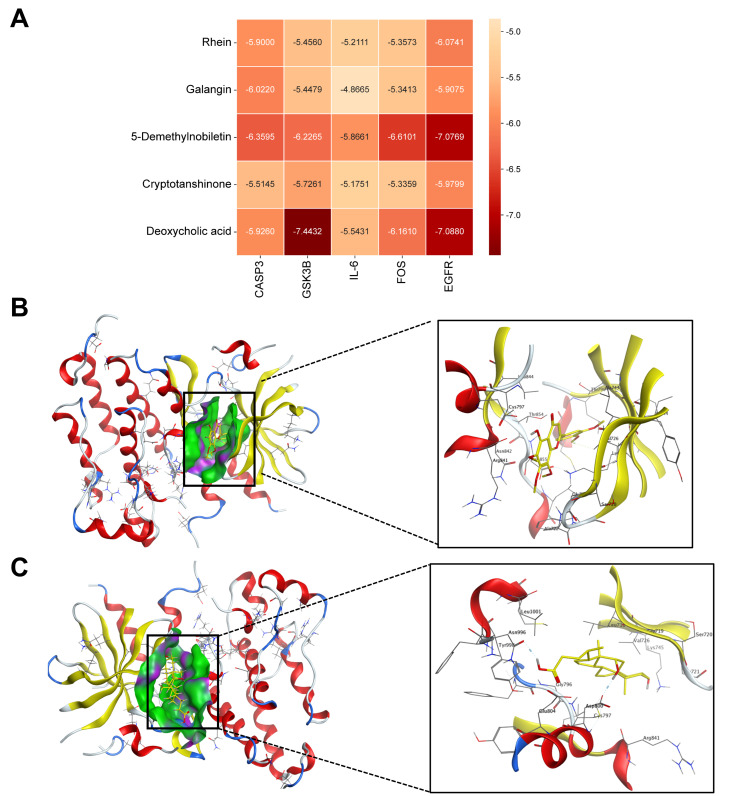

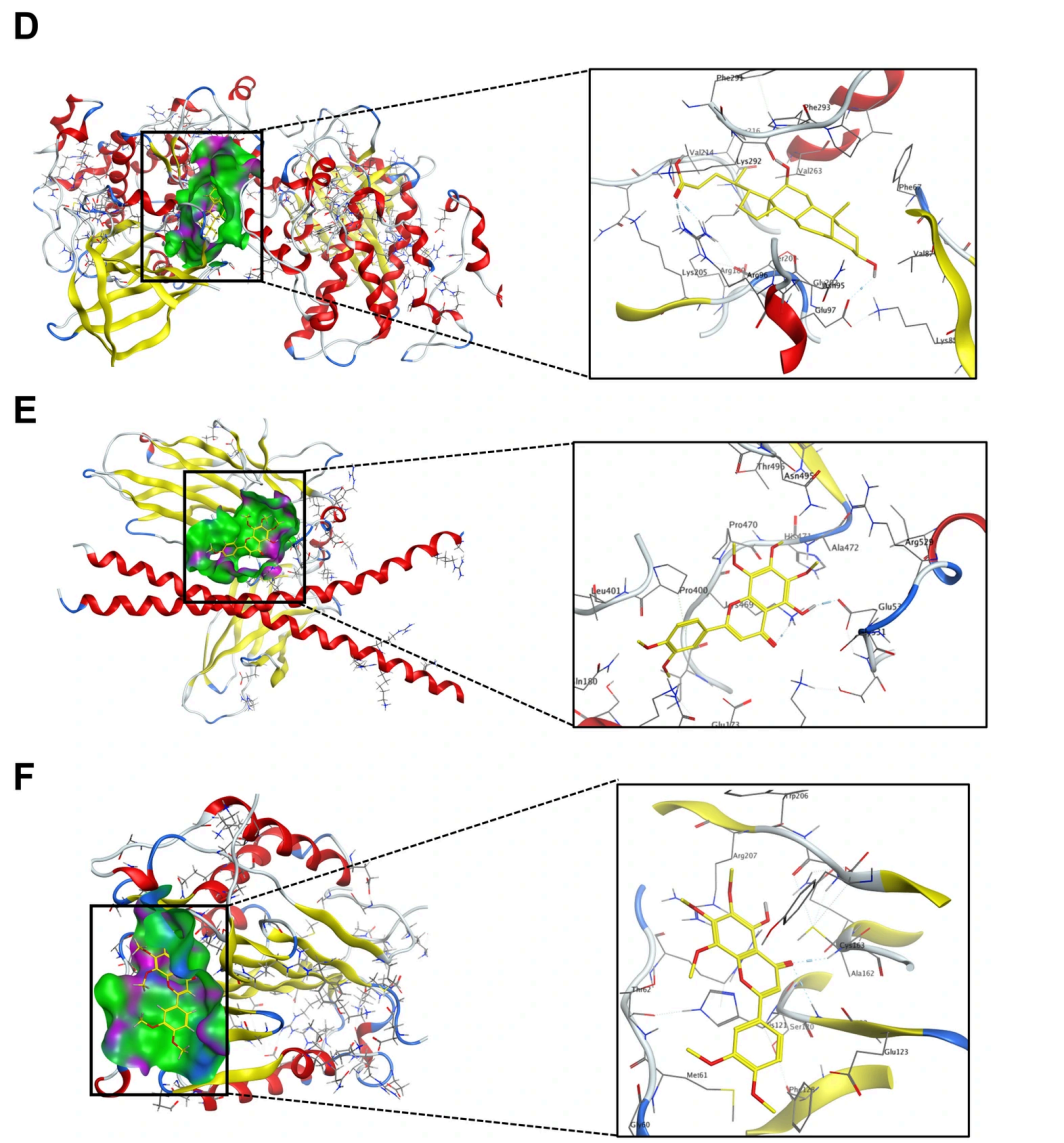
Molecular docking validation of key RSNPs constituents with hub targets. (**A**) Binding affinity heatmap. Color intensity corresponds to the predicted binding energy (kcal·mol^−1^), with darker colors indicating stronger binding affinity (lower energy). Dashed boxes highlight interactions with high binding affinity. (**B**–**F**) Detailed binding poses of representative complexes: (**B**) 5-Demethylnobiletin-EGFR, (**C**) Deoxycholic acid-EGFR, (**D**) Deoxycholic acid-GSK3B, (**E**) 5-Demethylnobiletin-FOS, and (**F**) 5-Demethylnobiletin-CASP3. Hydrogen bonds are shown as yellow dashed lines, and π–π stacking interactions as green dashed lines.

Molecular docking simulations were performed to evaluate the interactions between five key RSNPs constituents (cryptotanshinone, galangin, 5-demethylnobiletin, rhein, and deoxycholic acid) and five hub targets relevant to AKI (IL-6, CASP3, GSK3B, EGFR, and FOS) (Figure 4A). Binding affinities below −4.25 kcal·mol^−1^ are indicative of detectable interaction, values below −5.0 kcal·mol^−1^ reflect moderate binding, and those below −7.0 kcal·mol^−1^ represent high-affinity binding. Notably, most of the RSNP component–target complexes exhibited binding energies lower than −5.0 kcal·mol^−1^, demonstrating favorable molecular interactions between these bioactive compounds and critical AKI-related targets. Ligand–target interaction analysis (Figure 4B–F) showed that 5-Demethylnobiletin bound EGFR via H-bonds to Met769/Thr830 and π–π stacking with Phe699 (−8.2 kcal/mol). Deoxycholic acid bound EGFR through hydrophobic contacts with Leu694/Val702/Ala719 and an H-bond to Lys721, and bound GSK3B via H-bonds to Val135/Asp200 and hydrophobic contacts with Ile62/Leu132/Cys199 (−7.5 kcal/mol). 5-Demethylnobiletin interacted with FOS via π–π stacking with Phe154/Phe157 and H-bonds to Arg155/Glu168, and with CASP3 via H-bonds to Arg207/Ser236 and hydrophobic contacts with Trp206/Phe250/Tyr197 (−7.8 kcal/mol). These results provide computational validation of the potential therapeutic bioactivity of RSNPs constituents.

### 3.7. Characterization of RSNPs

**Figure 5:**
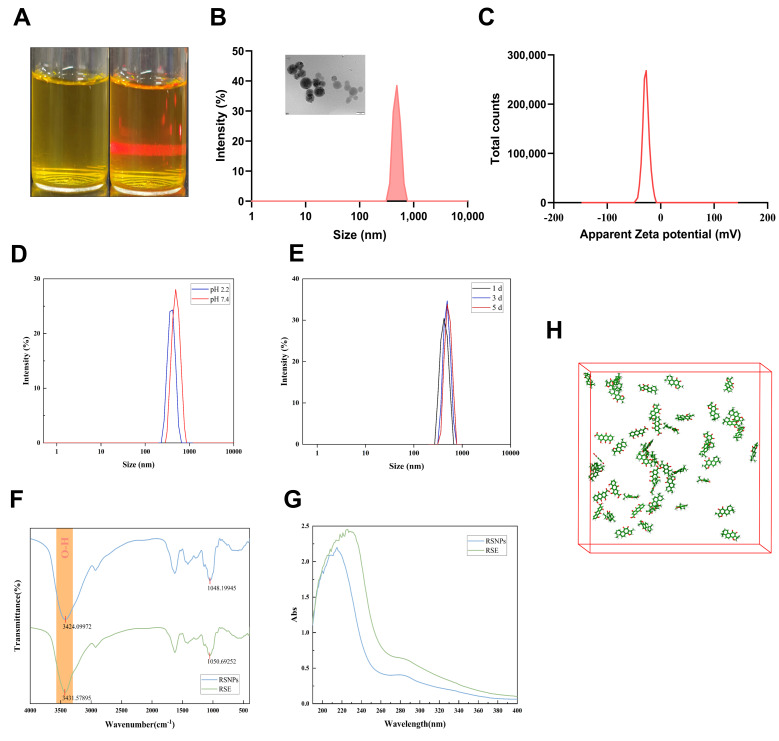

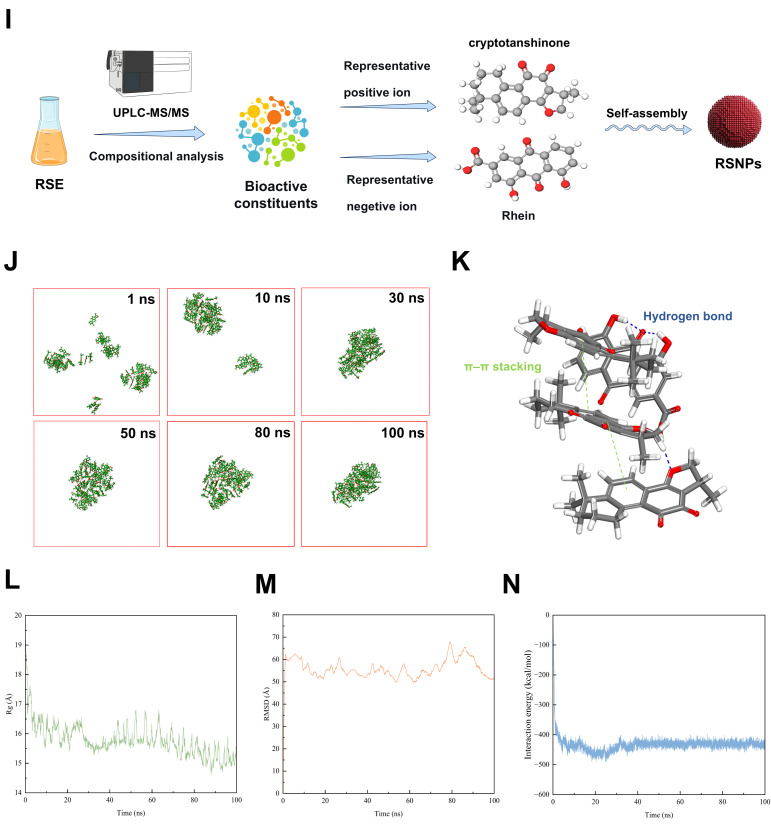
Nanoparticle characterization, stability, and self-assembly mechanism of RSNPs. (**A**–**G**) Experimental characterization: (**A**) Photograph of the Tyndall effect in RSNPs aqueous dispersion. (**B**) Hydrodynamic size distribution (left) and TEM image (right) of RSNPs. Scale bar: 200 nm. (**C**) Zeta potential distribution of RSNPs. Hydrodynamic diameter and zeta potential were measured in triplicate, and data are presented as mean ± SD (*n* = 3). (**D**) Characterization of RSNPs after incubation in buffers at different pH values. (**E**) Characterization of RSNPs after storage at 4 °C for different time periods. Each time point represents the mean ± SD of three independent replicate measurements. (**F**) UV-Vis absorption spectra of RSNPs. (**G**) FTIR spectra of RSNPs. The broadened and redshifted O–H stretching band at 3424 cm^−1^ suggests enhanced hydrogen bonding in RSNPs. (**H**–**N**) Molecular dynamics simulations: (**H**) Initial conformation for the self-assembly molecular dynamics simulation. (**I**–**K**) Conformational evolution of the simulation system at 1, 10, 30, 50, 80, and 100 ns. (**L**) Time evolution trajectory of the radius of gyration (Rg). (**M**) Time evolution trajectory of the root mean square deviation (RMSD). (**N**) Time evolution trajectory of the interaction energy.

The colloidal stability of RSNPs in aqueous dispersion was confirmed by a clear Tyndall effect (Figure 5A). Characterization of their physical properties showed a monodisperse hydrodynamic diameter of 500.33 ± 61.75 nm with a PDI of 0.278, and a zeta potential of −27.59 ± 6.2 mV (Figure 5B,C), collectively reflecting outstanding colloidal stability and homogeneity. TEM images further revealed uniformly spherical nanoparticles with well-defined morphology. Importantly, RSNPs retained structural integrity under physiologically relevant pH conditions (2.4–7.4), indicating strong resistance to gastrointestinal environments and highlighting their potential for oral delivery. Additional stability tests under refrigerated storage further supported the robustness of the nanoparticle formulation (Figure 5D,E). Together, these results confirm that phytoconstituents from rhubarb and *Salvia miltiorrhiza* undergo spontaneous self-assembly during decoction, forming nanoparticles with excellent pharmaceutical stability.

Spectroscopic analyses provided further insight into the self-assembly process. FTIR spectra displayed a broadened and redshifted hydroxyl stretching band at 3424 cm^−1^, indicating enhanced hydrogen bonding. Meanwhile, UV-Vis spectroscopy revealed a hypsochromic shift with reduced absorption intensity, consistent with π-π stacking-driven H-aggregation. These spectral features support a cooperative self-assembly mechanism in which hydrogen bonding and π-π interactions work synergistically to form a highly ordered nanostructure. This well-defined architecture offers a physicochemical basis for the effective drug delivery performance of RSNPs in AKI treatment.

### 3.8. Molecular Dynamics Elucidation of RSNPs Self-Assembly Mechanism

Cryptotanshinone and rhein were selected for molecular dynamics simulations based on their high mass fraction in the nanoparticle system, their role as core bioactive constituents in their respective source materials, and their structural predisposition for driving self-assembly. The simulations elucidated the molecular-level mechanism of RSNPs’ spontaneous self-assembly, revealing a well-defined hierarchical pathway. During the 100 ns simulation, the system—composed of 10 rhein molecules and 38 cryptotanshinone molecules—progressed through three distinct stages: initial nucleation (0–10 ns), characterized by molecular clustering; structural consolidation (10–30 ns), marked by hydrophobic core formation; and equilibrium stabilization (30–100 ns), leading to mature core–shell architectures (Figure 5J).

The assembly is governed by complementary non-covalent interactions. Cryptotanshinone molecules constitute the hydrophobic core through parallel π–π stacking, while polar functional groups guide the organization of the hydrophilic shell via extensive hydrogen bonding networks. These structural features are consistent with spectral data, confirming the intermolecular interactions driving the process. Quantitative analyses further corroborate the thermodynamic stability of the assemblies. The convergence of the root-mean-square deviation (RMSD < 0.2 nm after 30 ns) and a decreasing radius of gyration (Rg~2.1 nm) indicates structural compaction and uniformity (Figure 5L,M), aligning with experimental DLS measurements. A sustained interaction energy of −52.3 kcal/mol affirms the spontaneity of the process and helps explain the nanoparticles’ robust stability under varying pH conditions (Figure 5N). By integrating computational modeling with experimental validation, this multiscale approach establishes a robust framework for the rational design of natural product-based nanotherapeutics with controlled architectures.

### 3.9. Evaluation of the Antioxidant Capacity of RSNPs

**Figure 6:**
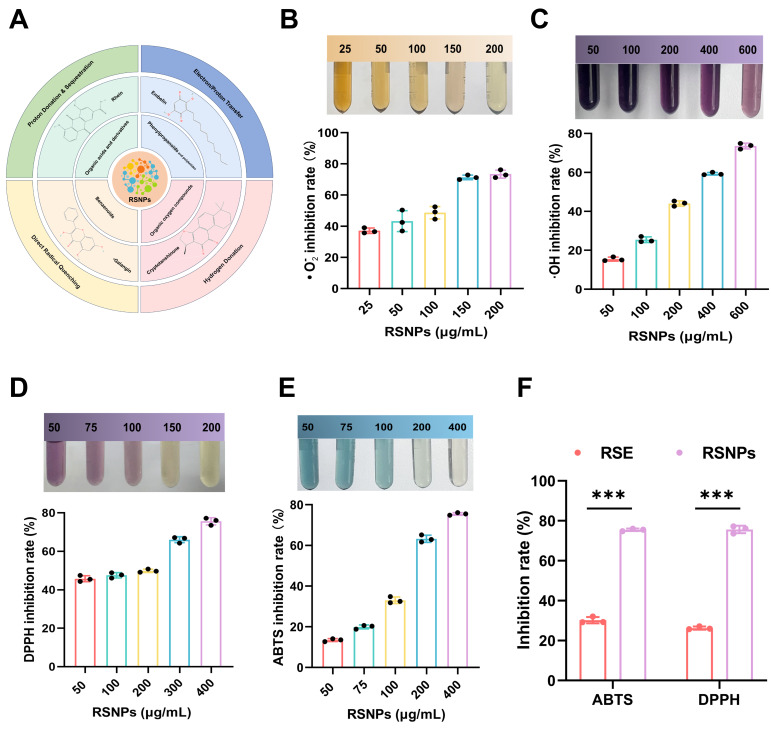
In vitro antioxidant activity evaluation of RSNPs. (**A**) Schematic diagram of RSNPs scavenging four radical species. (**B**) Dose-dependent scavenging curve of RSNPs against superoxide anions (•O_2_^−^) (IC50 = 231.6 ± 5.9 μg/mL). (**C**) Dose-dependent scavenging curve of RSNPs against hydroxyl radicals (•OH) (IC50 = 231.6 ± 5.9 μg/mL). (**D**,**E**) Scavenging efficiency of RSNPs against DPPH (IC50 = 196.9 ± 7.2 μg/mL) and ABTS^+^ radicals (IC50 = 157.8 ± 4.3 μg/mL). (**F**) Comparative scavenging capacity of RSNPs and RSE against DPPH and ABTS^+^ radicals. *** *p* < 0.001.

RSNPs exhibited notable dose-dependent scavenging capacity against all four radical species tested. A schematic representation of the four radical species scavenged by RSNPs is shown in Figure 6A. At a concentration of 200 μg/mL, RSNPs eliminated approximately 75% of superoxide anions (•O_2_^−^) (Figure 6B), while a concentration of 600 μg/mL resulted in about 73% scavenging of hydroxyl radicals (•OH) (Figure 6C). Furthermore, treatment with 400 μg/mL RSNPs led to a roughly 75% reduction in both DPPH and ABTS^+^ radicals (Figure 6D,E), with all reaction systems reaching kinetic equilibrium within 30 min. Notably, under equivalent concentration conditions, RSNPs demonstrated significantly superior scavenging activity against DPPH and ABTS^+^ radicals compared to the precursor extract RSE (Figure 6F), indicating that nanostructural reorganization not only preserved but also enhanced the antioxidant activity.

This enhanced performance is attributed to the synergistic molecular assembly within RSNPs at the nanoscale. Specifically, spatially ordered ortho-dihydroxyl flavonoids facilitate targeted •OH scavenging via efficient hydrogen atom transfer, while conjugated phenylpropanoids contribute to •O_2_^−^ quenching through electron delocalization. Simultaneously, catechol-based organic acids neutralize cationic radicals via a proton-coupled electron transfer mechanism. Critically, the nano-confined environment creates a cooperative interface that integrates these distinct pathways, enabling synergistic interactions and establishing an efficient electron transfer network that surpasses the simple additive effects of individual components. This integrated mechanism allows for broad-spectrum and synergistic radical scavenging.

### 3.10. Evaluation of In Vitro Absorption Characteristics

Cryptotanshinone and rhein were selected as marker compounds because they are the most abundant bioactive constituents in RSNPs and are known to exhibit poor oral bioavailability, making them ideal candidates for evaluating absorption enhancement by the nanoformulation. The intestinal absorption behaviors of cryptotanshinone and rhein were compared between RSNPs and crude RSE using the rat everted gut sac model. Both compounds showed concentration-dependent and region-specific absorption patterns, with RSNPs exhibiting consistently superior delivery performance.

**Figure 7:**
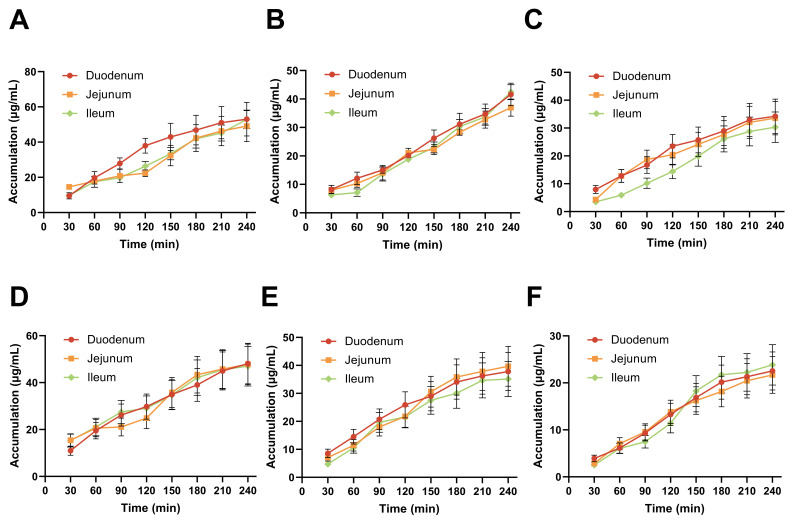

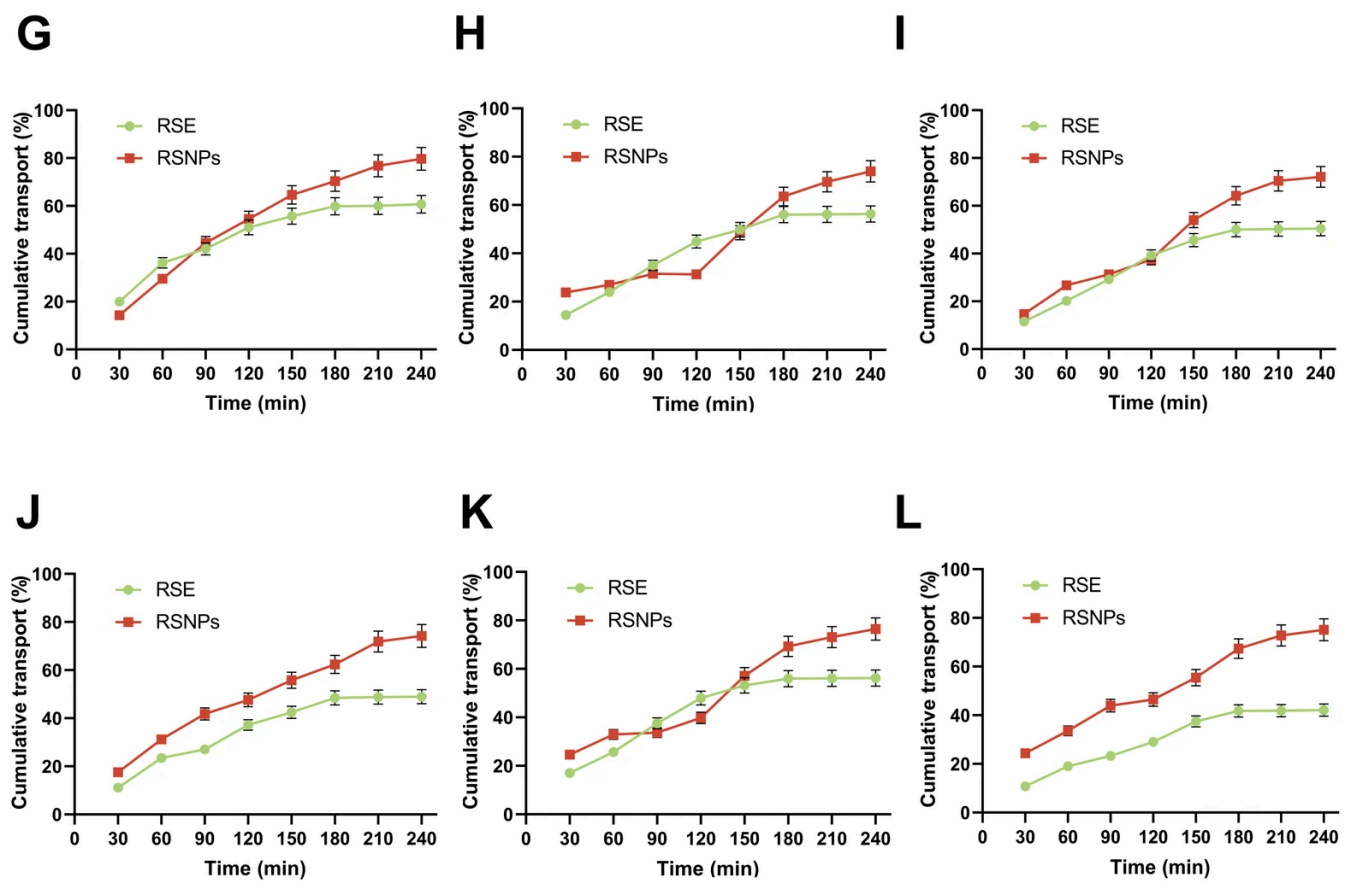
Intestinal absorption characteristics of cryptotanshinone and rhein in RSNPs (Using rat everted gut sac model). Data represent mean values from four independent biological samples (*n* = 4). (**A**–**C**) Intestinal absorption of cryptotanshinone from RSNPs: Cumulative absorption in (**A**) duodenum, (**B**) jejunum, and (**C**) ileum at different concentrations (5–10 μg/mL). (**D**–**F**) Intestinal absorption of rhein from RSNPs: Cumulative absorption in (**D**) duodenum, (**E**) jejunum, and (**F**) ileum at different concentrations (5–10 μg/mL). (**G**–**I**) Transport efficiency comparison of cryptotanshinone: RSNPs vs. RSE in (**G**) duodenum, (**H**) jejunum, and (**I**) ileum. (**J**–**L**) Transport efficiency comparison of rhein: RSNPs vs. RSE in (**J**) duodenum, (**K**) jejunum, and (**L**) ileum.

Accumulation of both compounds increased in the duodenum, jejunum, and ileum as concentrations rose from 5 to 10 mg/mL (Figure 7A,B). Cryptotanshinone displayed sustained-release properties, with rapid initial accumulation during 0–120 min followed by markedly slowed absorption rates—suggesting either controlled release behavior or potential transporter saturation. In contrast, rhein absorption occurred more rapidly, reaching near-maximal levels within 60 min and plateauing by 120 min, indicating either enhanced permeability or faster release from the nanocarrier. Regional analysis identified the duodenum as the primary absorption site for both formulations, showing higher accumulation levels and rates than the jejunum and ileum. Notably, rhein showed better accumulation in jejunal and ileal regions at medium and high concentrations (7.5 and 10 mg/mL), implying distinct transport mechanisms that may involve regional differences in efflux transporter expression or paracellular permeability. RSNPs demonstrated comprehensive advantages in transport efficiency over RSE. The nanoparticles enhanced transmembrane transport in the duodenum, as reflected by steeper cumulative transport percentage-time curves. In distal intestinal regions, RSNPs particularly improved cryptotanshinone transport in the ileum and enhanced rhein absorption in the jejunum, effectively overcoming regional absorption barriers for both compounds (Figure 7C,D).

Based on the collective evidence, RSNPs appear to enhance intestinal absorption of both poorly soluble compounds through multiple pathways, including solubility improvement, mucoadhesive properties, and tailored release kinetics. These insights help advance the development of nanoparticle-based oral delivery systems for traditional Chinese medicine compounds.

### 3.11. Prophylactic Nephroprotective Efficacy of RSNPs In Vivo

Based on the renal-targeting characteristics of RSNPs, we further assessed their prophylactic efficacy in an established mouse model of CDDP-induced nephrotoxicity. Mice were pretreated for 7 days with low-dose (1.0 g/kg) or high-dose (1.5 g/kg) RSNPs, using conventional RSE (1.5 g/kg) as a reference control, before receiving a single nephrotoxic dose of CDDP (15 mg/kg, i.p.).

**Figure 8:**
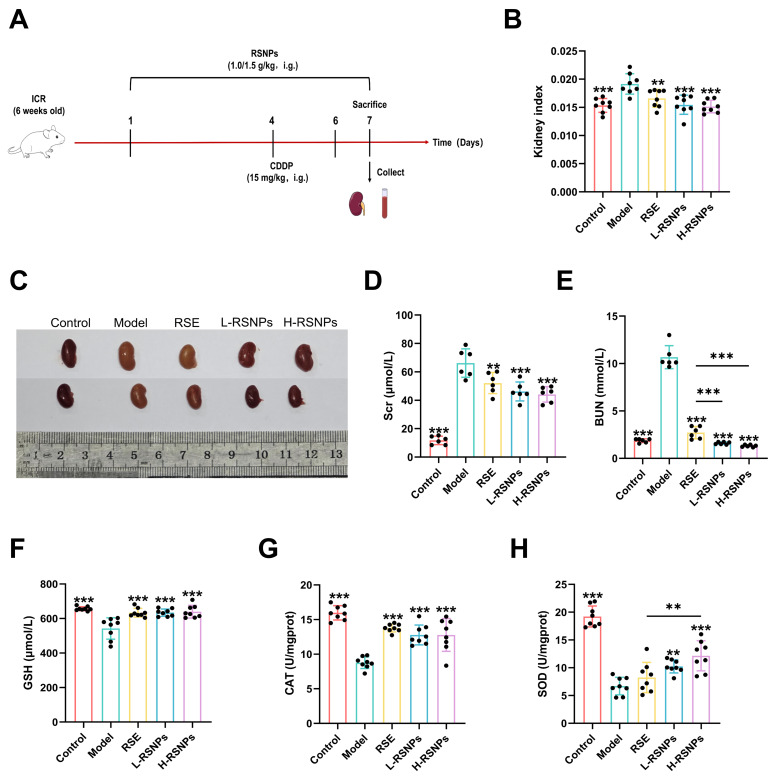

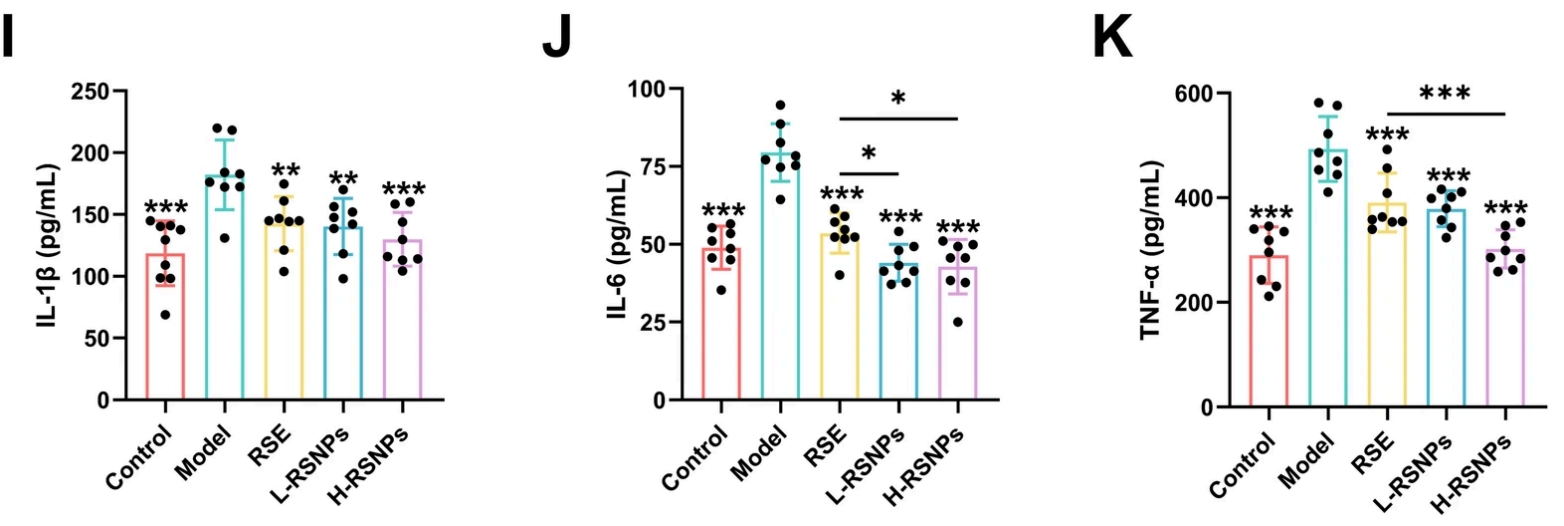

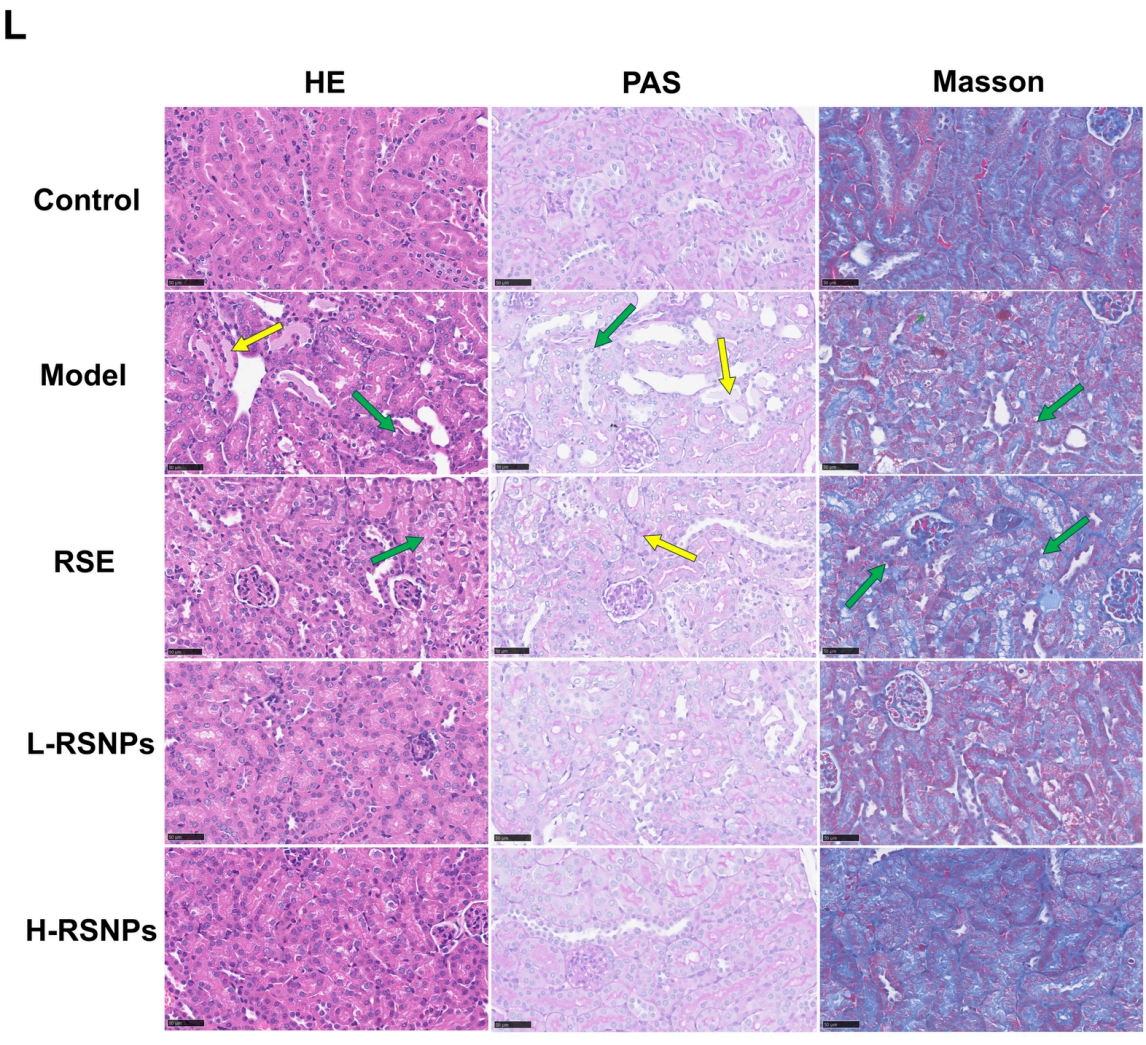
Prophylactic protective effects of RSNPs against cisplatin-induced nephrotoxicity. (**A**) Schematic diagram of experimental design. (**B**) Kidney index comparison among groups. (**C**) Serum creatinine levels. (**D**) Blood urea nitrogen levels. (**E**) Glutathione content in renal tissue. (**F**–**H**) Antioxidant enzyme activities in renal tissue. (**I**–**K**) Inflammatory cytokine levels. (**L**) Renal histopathology by H&E, PAS, and Masson’s trichrome staining (scale bar = 50 μm). The yellow arrow indicates the protein tube type, and the green arrow shows the renal epithelial vacuoles. Data are presented as mean ± SD, * *p* < 0.05, ** *p* < 0.01, and *** *p* < 0.001.

Prophylactic administration of RSNPs provided comprehensive protection against renal injury. Macroscopic examination showed that RSNPs, particularly at the high dose, markedly alleviated CDDP-induced pathological changes such as kidney enlargement and tissue pallor (Figure 8C). Functional analyses further demonstrated that RSNPs dose-dependently reduced the elevated kidney index and normalized serum creatinine and blood urea nitrogen levels, with high-dose RSNPs consistently outperforming the conventional RSE (Figure 8D,E).

At the physiological level, RSNPs effectively preserved renal homeostasis by counteracting CDDP-induced oxidative imbalance. Treatment restored the activities of key antioxidant enzymes and reversed glutathione depletion in a dose-dependent manner (Figure 8F–H). In parallel, RSNPs robustly suppressed the CDDP-triggered increase in pro-inflammatory mediators (TNF-α, IL-1β, IL-6), with high-dose RSNPs showing the strongest anti-inflammatory effect (Figure 8I–K). Histopathological evaluation provided structural confirmation of this protection: H&E and PAS staining revealed that RSNPs pretreatment significantly attenuated tubular injury, including epithelial degeneration and cast formation, in a dose-related manner. Masson’s trichrome staining further demonstrated preserved basement membrane architecture, indicating structural integrity was maintained against CDDP-induced damage (Figure 8L).

In summary, prophylactic RSNPs treatment conferred multi-faceted protection against CDDP nephrotoxicity by coordinately maintaining redox balance, modulating inflammatory responses, and preserving renal microstructure. The clear dose–response relationship and consistent superiority over conventional RSE highlight the translational potential of RSNPs as a promising nephroprotective strategy.

### 3.12. Protective Effects of RSNPs In Vitro

**Figure 9:**
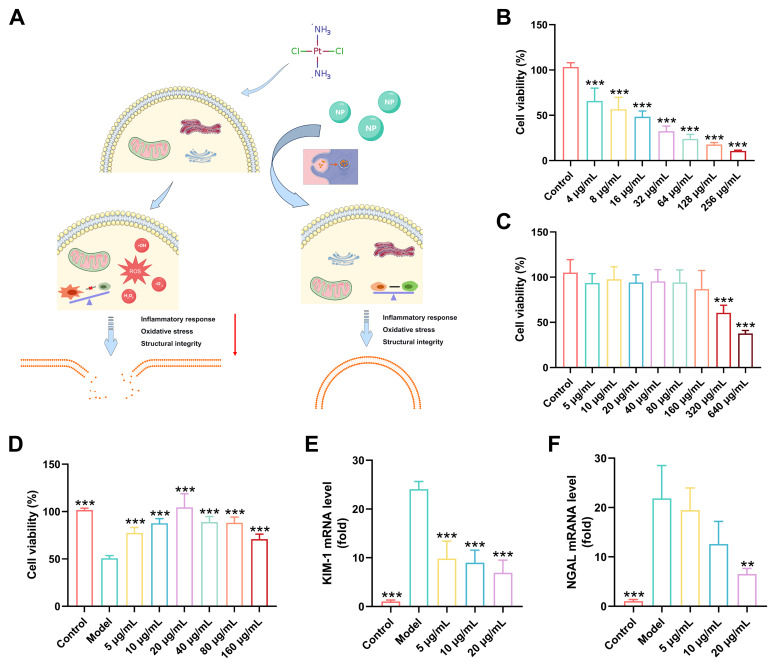

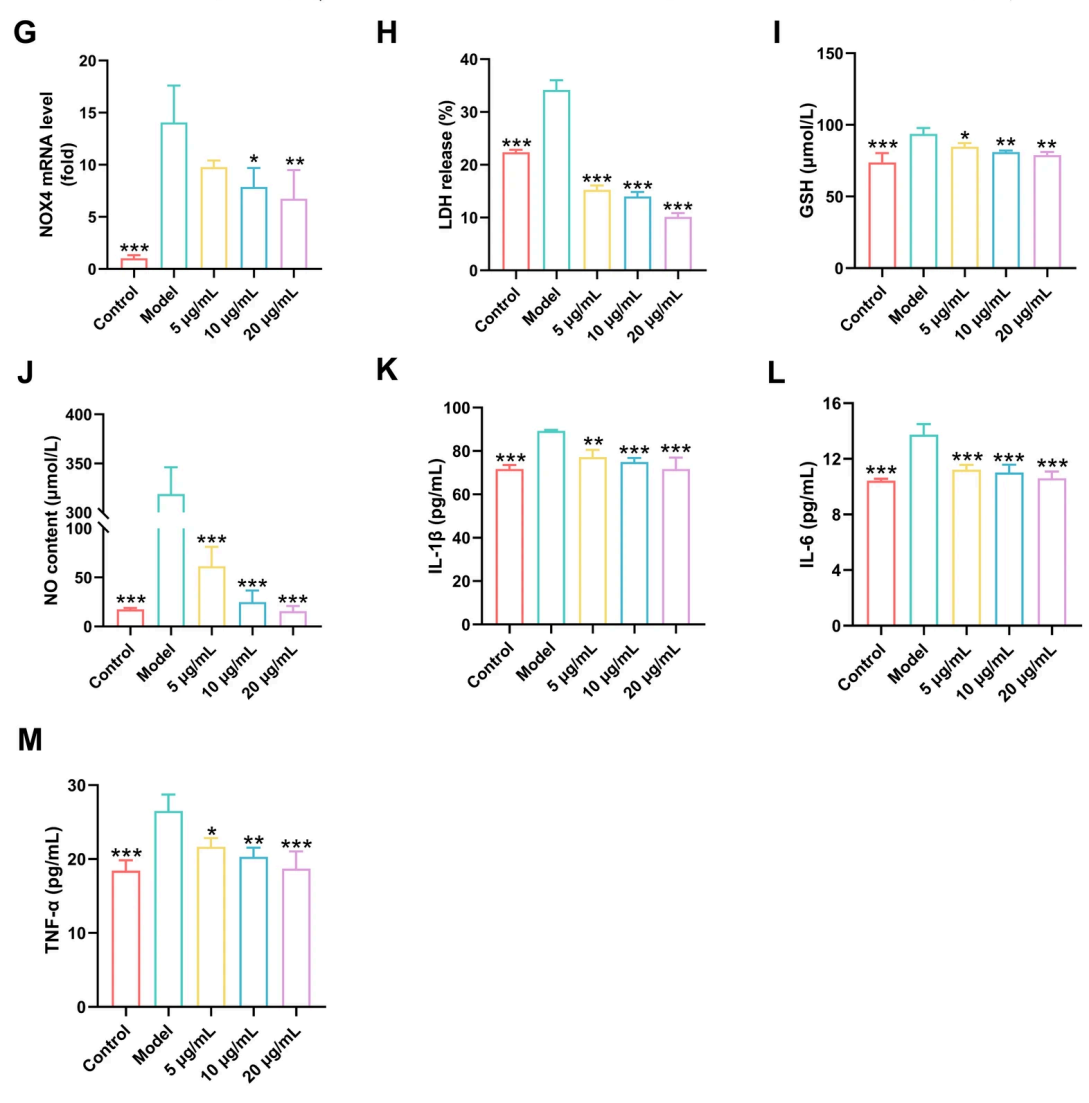
Protective effects of RSNPs against cisplatin-induced renal tubular epithelial cell injury. (**A**) Schematic diagram illustrating the renoprotective mechanisms of RSNPs. (**B**) Dose–response curve of cisplatin-induced cellular injury. (**C**) Biocompatibility assessment of RSNPs at various concentrations. (**D**) Protective effect of RSNPs pretreatment on cell viability after cisplatin challenge. (**E**,**F**) Effects of RSNPs on renal injury biomarkers KIM-1 and NGAL mRNA expression. (**G**) Regulatory effect of RSNPs on NOX4 mRNA expression. (**H**) Inhibitory effect of RSNPs on LDH release. (**I**) Modulatory effect of RSNPs on glutathione homeostasis. (**J**–**M**) Inhibitory effects of RSNPs on pro-inflammatory cytokines (IL-1β, IL-6, TNF-α) and NO secretion. Data are presented as mean ± SD, * *p* < 0.05, ** *p* < 0.01, *** *p* < 0.005.

We next evaluated the renoprotective effects of RSNPs against CDDP-induced injury in renal tubular epithelial cells. A dose–response study first established 8 μg/mL CDDP as an optimal concentration for inducing consistent cellular damage (Figure 9B). Prior to protection assays, RSNPs were confirmed to exhibit no detectable cytotoxicity across a broad concentration range (5–160 μg/mL), supporting their biocompatibility and therapeutic applicability (Figure 9C). Pretreatment with RSNPs effectively preserved cell viability in a concentration-dependent manner, with nearly complete restoration of cellular homeostasis observed at 20 μg/mL following CDDP challenge (Figure 9D).

At the molecular level, CDDP significantly upregulated transcripts of the renal injury biomarkers KIM-1 and NGAL (Figure 9E,F), whereas RSNPs administration suppressed their expression in a graded fashion. Maximal inhibition occurred at 20 μg/mL, indicating targeted protection against tubular epithelial injury. We also observed that CDDP induced overexpression of NADPH oxidase 4 (NOX4) (Figure 9G), implicating oxidative mechanisms in the injury process. This effect was concentration-dependently attenuated by RSNPs, with near-complete suppression at 20 μg/mL, supporting a role for redox modulation in RSNP-mediated protection.

CDDP also disrupted membrane integrity, as reflected by increased LDH release and altered glutathione homeostasis (Figure 9H,I). Both parameters were effectively stabilized by RSNPs pretreatment, again most prominently at 20 μg/mL, underscoring membrane protection as a key component of the cytoprotective response. Furthermore, RSNPs potently inhibited the secretion of pro-inflammatory cytokines (IL-1β, IL-6, and TNF-α) and NO in a dose-dependent manner (Figure 9J–M), with maximal suppression at 20 μg/mL. These results establish a clear link between anti-inflammatory activity and the overall cytoprotective efficacy of RSNPs.

### 3.13. RSNPs Suppress CDDP-Induced Apoptosis

CDDP-induced nephrotoxicity is largely driven by DNA damage-mediated activation of the p53 signaling pathway, which ultimately triggers mitochondrial-dependent apoptosis. In this study, we assessed the anti-apoptotic potential of RSNPs by combining transcriptional profiling of key apoptotic genes with quantitative analysis of cell death progression.

**Figure 10:**
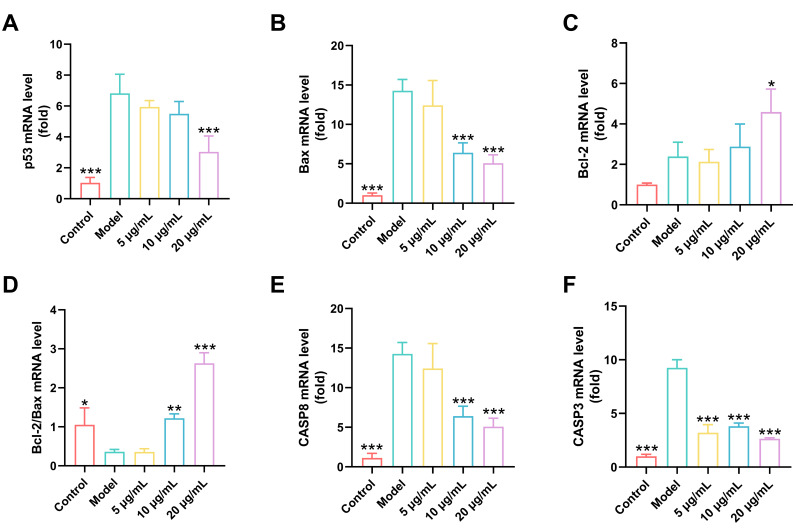

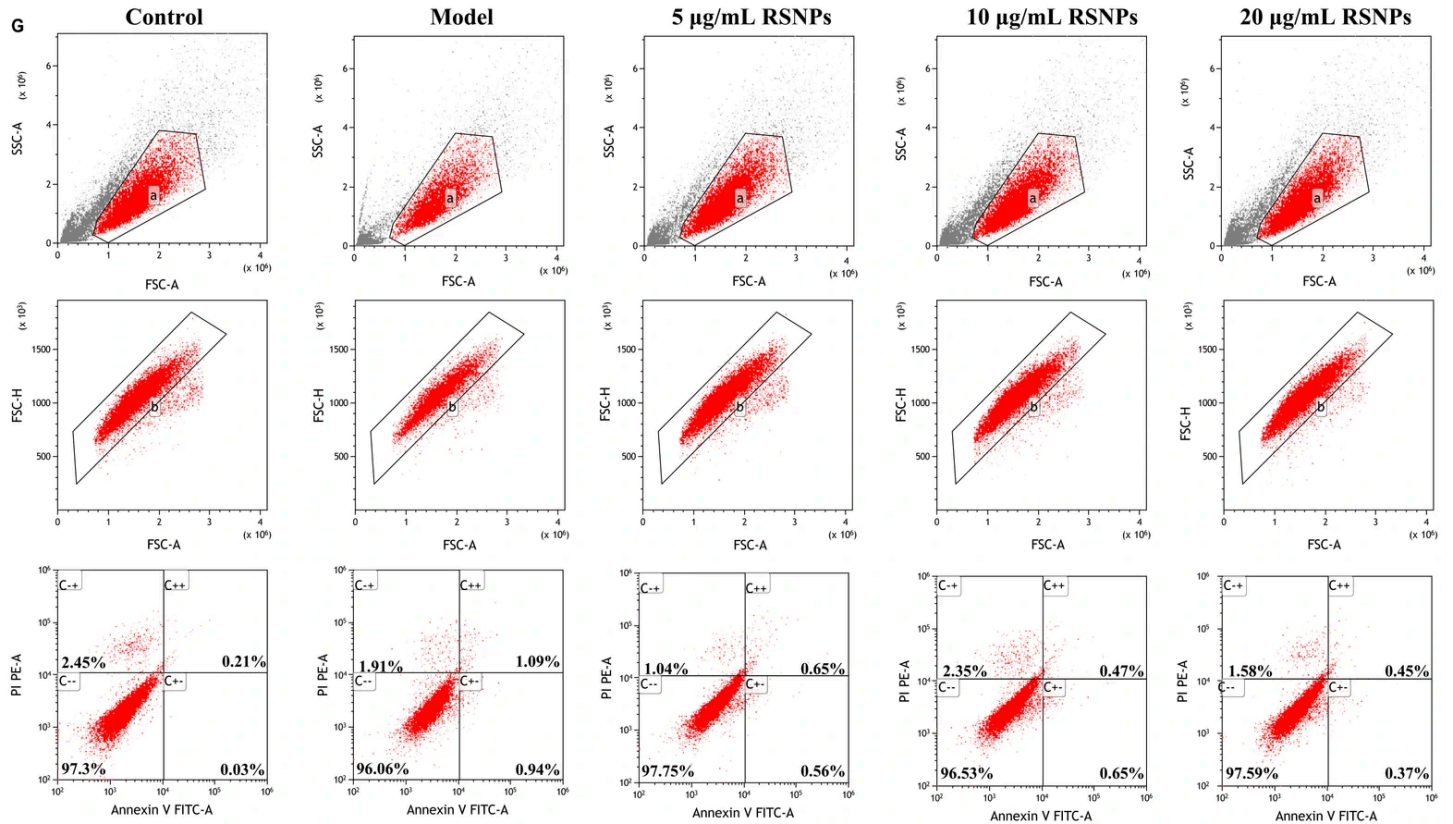
Protective mechanism of RSNPs against cisplatin-induced apoptosis. (**A**) p53 mRNA expression levels. (**B**) Bcl-2 mRNA expression levels. (**C**) Bax mRNA expression levels. (**D**) Bcl-2/Bax ratio. (**E**) CASP8 mRNA expression levels. (**F**) CASP3 mRNA expression levels. (**G**) Apoptosis analysis by flow cytometry. (a) Viable cells were gated based on FSC/SSC characteristics; (b) Single cells were selected by FSC-A/FSC-H gating to exclude doublets; (c) Apoptosis was assessed by Annexin V-FITC/PI staining. Data are presented as mean ± SD, * *p* < 0.05, ** *p* < 0.01, *** *p* < 0.001.

Upon CDDP exposure, a coordinated apoptotic cascade was activated, marked by elevated p53 transcript levels and subsequent disruption of Bcl-2 family dynamics (Figure 10A–D). Specifically, we detected increased expression of the pro-apoptotic protein Bax alongside downregulation of the anti-apoptotic factor Bcl-2, leading to a markedly reduced Bcl-2/Bax ratio. These changes further propagated through the apoptotic cascade, as indicated by upregulation of both initiator (CASP8) and executioner (CASP3) caspases at the mRNA level (Figure 10E,F). Flow cytometric analysis using annexin V/propidium iodide staining quantitatively confirmed these observations, showing a clear increase in early and late apoptotic cell populations and a corresponding decline in viable cells following CDDP treatment (Figure 10G).

Pre-treatment with RSNPs, however, conferred substantial protection against CDDP-triggered apoptosis through multi-layered regulatory mechanisms. In a concentration-dependent manner, RSNPs suppressed p53 transcriptional activation, restored the balance between Bcl-2 and Bax, and dampened the induction of the caspase cascade. Flow cytometry further validated these effects, demonstrating that RSNPs reduced apoptotic cell populations while increasing the proportion of viable cells. Maximal protection was observed at 20 μg/mL RSNPs, which effectively restored apoptotic gene expression to near-normal levels and preserved cellular integrity.

## 4. Discussion

The modernization of Traditional Chinese Medicine requires a fundamental shift in perspective—from studying isolated chemical constituents to understanding the integrated functional assemblies that form naturally during herbal preparation [22]. In this work, we report a previously unrecognized phenomenon: the spontaneous formation of self-assembled nanoparticles in *Rheum palmatum* L.–*Salvia miltiorrhiza* decoction. These RSNPs represent functional supramolecular assemblies with intrinsic therapeutic properties, challenging the conventional view of TCM extracts as simple mixtures of molecular compounds. By integrating computational modeling, advanced spectroscopy, and biological validation, we have delineated the hierarchical organization of RSNPs and their multifaceted mechanisms of action against acute kidney injury, offering new nanoscale insights into TCM pharmacology.

For oral nanomedicines, therapeutic efficacy depends critically on stability throughout the gastrointestinal environment [23]. RSNPs exhibit excellent colloidal properties, including a monodisperse size distribution (PDI = 0.28), a substantial negative surface charge (−27.6 mV), and a well-defined spherical morphology. Notably, they maintain structural integrity across a physiologically relevant pH range (2.4–7.4), outperforming many synthetic nanocarriers that tend to aggregate under gastric conditions [24]. This robust stability arises from a sophisticated network of non-covalent interactions—primarily hydrogen bonding and π-π stacking—that collectively stabilize the nanoparticle architecture [25,26]. We propose that this highly organized structure emerges naturally during traditional decoction, representing an evolutionarily optimized delivery system capable of retaining its functional conformation during gastrointestinal transit.

Whereas contemporary nanomedicine predominantly focuses on single-component or binary systems [27,28], our investigation reveals a more intricate self-assembly phenomenon: under suitable thermodynamic conditions, over one hundred structurally diverse phytochemicals spontaneously organize into highly ordered nanostructures with monodisperse characteristics. Our spectroscopic evidence aligns with established supramolecular characterization methodologies reported in contemporary literature. The observed hydrogen bonding patterns [29] and π-π stacking phenomena [30] correspond closely with documented non-covalent interactions in self-assembling systems, particularly those involving polyphenolic compounds and aromatic structures [31]. Complementary evidence from Fourier-transform infrared spectroscopy and ultraviolet–visible absorption spectroscopy substantiates this supramolecular organization—the former demonstrates significantly enhanced hydrogen bonding networks, while the latter exhibits characteristic π-π stacking-induced H-aggregation signatures. These findings not only confirm the feasibility of multilevel molecular coordination in complex systems but also establish a new design paradigm for developing natural component-based nanomedicines.

Molecular dynamics simulations unraveled the dynamic self-assembly mechanism with unprecedented clarity. Our 100 ns trajectory analysis of a 48-molecule system (10 rhein/38 cryptotanshinone) revealed a well-orchestrated three-stage pathway: molecular diffusion and initial clustering during nucleation (0–10 ns), progressive aromatic stacking core formation in the consolidation phase (10–30 ns), and ultimate structural equilibration (30–100 ns). These kinetic profiles closely align with Feng et al.’s [32] berberine assembly system, while demonstrating enhanced organizational sophistication through multi-component coordination. The core–shell architecture exhibited remarkable thermodynamic stability, quantified through converging RMSD values (<0.2 nm after 30 ns), stable radius of gyration (~2.1 nm), and consistent interaction energy (−52.3 kcal/mol). These structural characteristics prove comparable to molecular dynamics simulations by Liu et al. [33] and Rosi et al. [34], yet demonstrate a clear advantage in structural stability conferred by molecular diversity. Traditional approaches appear to have underestimated the emergent properties generated through multi-component synergies [35]. This work establishes that natural compounds inherently possess the molecular blueprint for constructing stable, hierarchically ordered nanostructures without synthetic intervention.

The enhanced therapeutic efficacy of purified RSNPs over the crude extract (RSE) stems from improved oral bioavailability and multi-target engagement. A key factor is the hydrodynamic diameter of RSNPs (~500 nm), which optimally balances gastrointestinal stability with efficient cellular uptake [36]. This size range not only prolongs intestinal retention but may also enhance clathrin- and caveolae-mediated endocytosis, thereby overcoming the absorption constraints commonly associated with molecular dispersions [37,38]. Experimental data from the everted gut sac model demonstrate that RSNPs significantly enhance the intestinal absorption efficiency of both cryptotanshinone and rhein, as evidenced by markedly increased cumulative transport compared to conventional extracts. Though the precise absorption mechanisms remain to be fully elucidated, the unique physicochemical properties of the nano-formulation—including optimized size distribution, improved solubility, and potential interfacial interactions with the intestinal mucosa—may collectively contribute to this absorption enhancement [39,40]. These findings provide compelling experimental evidence for investigating the bioavailability-promoting effects of self-assembled nano-systems derived from traditional herbal medicine.

Beyond delivery advantages, RSNPs exhibit sophisticated polypharmacology, simultaneously modulating interconnected AKI pathways. Network analysis and molecular docking predict multi-target engagement with key nodes such as IL-6, CASP3, and EGFR. Experimental validation confirms coordinated regulation of oxidative stress, inflammatory cascades, and apoptotic signaling, with particular precision in maintaining Bcl-2/Bax balance and caspase activation thresholds. This system-level pharmacology mirrors modern combination therapy [41,42], yet avoids its pharmacokinetic complications through intrinsic coordination within a single nanoscale entity.

Although this work establishes RSNPs as a novel class of natural nanotherapeutics, several challenges remain. The precise “molecular code” governing self-assembly in such complex systems has yet to be fully deciphered. Future studies using single-particle analysis and synthetic biology approaches may uncover design principles for engineering enhanced natural nanoparticles. In addition, comprehensive biodistribution and long-term safety profiles warrant further investigation.

This study has several limitations. First, while time-dependent stability and molecular dynamics simulations strongly support that RSNPs are genuine self-assembled nanostructures, we did not perform direct impurity assays (e.g., protein or polysaccharide quantification) or single-particle elemental mapping (e.g., TEM-EDS). The purification method (differential centrifugation with multiple washes) effectively removes unbound small molecules, as confirmed by UPLC-MS/MS analysis of the final wash supernatant. Moreover, the observed bioactivities are consistent with the known properties of the identified phytochemicals, indirectly supporting the purity of the preparation. Nevertheless, future studies should include more comprehensive purity assessment and elemental analysis to further validate the composition of RSNPs.

The discovery of innate nanostructures in TCM decoctions points to a vast and unexplored landscape of natural nanomedicines. We envision a new research paradigm that systematically investigates self-assembling systems across traditional formulations, potentially yielding a diverse portfolio of nature-inspired nanotherapeutics. Such efforts could effectively bridge ancestral wisdom with modern nanotechnology, advancing both TCM modernization and innovative drug development.

## 5. Conclusions

This research indicates that the active components in the *Rheum palmatum* L.–*Salvia miltiorrhiza* decoction can spontaneously self-assemble through hydrogen bonding and π-π stacking into stable nanoparticles. These nanoparticles are endowed with remarkable antioxidant capacity, good intestinal absorption, and strong renal protective effects in vivo via multi-target synergistic mechanisms. Of course, a very important meaning of this work lies in the discovery of a novel natural nanomedicine, but more significantly, it was experimentally proved that “self-assembled nanostructure” can be one of the substantive bases of the effect of the herbal formula. This provides a fundamental shift in insight from a traditional “component-based” view to a modern “structure-based” one and thus offers a novel theoretical foundation for establishing complex formulations of traditional medicine.

## Figures and Tables

**Table 1:** Primer sequences for RT-PCR analysis.

Gene	GenBank Accession No.	Primer Sequence (5′–3′)
*KIM-1*	NM_001173393.3	Forward: ATTGTTGCCGTGTTGAGCAC Reverse: GTAGTCGTGACCTTGGGTGG
*NGAL*	XM_047423376.1	Forward: AACCAAGGAGCTGACTTCGG Reverse: GGTCGATTGGGACAGGGAAG
*Caspase3*	NM_001354777.2	Forward: TGCATACTCCACAGCACCTG Reverse: TCTGTTGCCACCTTTCGGTT
*Caspase8*	NM_001080124.2	Forward: GGAGGAGTTGTGTGGGGTAATG Reverse: CGAGGTTTGCTTTTCATTTGGT
*Bcl2*	NM_000633.3	Forward: GAACTGGGGGAGGATTGTGG Reverse: CATCCCAGCCTCCGTTATCC
*Bax*	NM_001291428.2	Forward: ATGGACGGGTCCGGGGAG Reverse: TTATGGAGGAAAAACACAGTCCA
*NOX4*	NM_001143836.3	Forward: TGACGTTGCATGTTTCAGGAG Reverse: AGCTGGTTCGGTTAAGACTGAT
*TP53*	NM_000546.6	Forward: CAGCACATGACGGAGGTTGT Reverse: TCATCCAAATACTCCACACGC
*β-actin*	NM_001101.5	Forward: GGGAAATCGTGCGTGACATT Reverse: GGAACCGCTCATTGCCAAT

## Data Availability

Datasets used or analyzed in this study are available from the corresponding author upon reasonable request.
